# AdMSC spheroids encapsulating antioxidant hybrid protein carrier for irradiation-damaged salivary gland repair

**DOI:** 10.1016/j.bioactmat.2026.03.049

**Published:** 2026-04-03

**Authors:** Byulhana Kim, Hyerin Yoo, Young Ju Son, Seyun Ahn, Jeonghoon Lee, Young Kim, Tae Hee Kim, Min Rye Eom, Young Bin Choy, Justin J. Chung, Seong Keun Kwon

**Affiliations:** a Interdisciplinary Program in Bioengineering, College of Engineering, Seoul National University, Seoul, 08826, Republic of Korea; b Department of Transdisciplinary Medicine, Seoul National University Hospital, Seoul, 03080, Republic of Korea; c Interdisciplinary Program in Stem Cell Biology, Seoul National University College of Medicine, Seoul, 03080, Republic of Korea; d Department of Otorhinolaryngology-Head and Neck Surgery, Biomedical Research Institute, Seoul National University Hospital, Seoul, 03080, Republic of Korea; e Department of Fusion Research and Collaboration, Biomedical Research Institute, Seoul National University Hospital, Seoul, 03080, Republic of Korea; f Innovative Medical Technology Research Institute, Seoul National University Hospital, Seoul 03080, Republic of Korea; g Department of Clinical Medical Sciences, Seoul National University College of Medicine, Seoul, 03080, Republic of Korea; h Institute of Medical and Biological Engineering, Medical Research Center, Seoul National University, Seoul, 03080, Republic of Korea; i Department of Biomedical Engineering, Seoul National University College of Medicine, Seoul, 03080, Republic of Korea; j Department of Medicine, Seoul National University College of Medicine, Seoul, 03080, Republic of Korea; k Department of Otorhinolaryngology-Head and Neck Surgery, College of Medicine, Seoul National University, Seoul, 03080, Republic of Korea; l Department of Otorhinolaryngology-Head and Neck Surgery, Seoul National University Hospital, Seoul, 03080, Republic of Korea; m Cancer Research Institute, Seoul National University, Seoul, 03080, Republic of Korea; n Sensory Organ Research Institute, Seoul National University Medical Research Center, Seoul, 03080, Republic of Korea

**Keywords:** Antioxidant hydrogel, Adipose-derived MSC spheroids, Xerostomia, Injectable hydrogel, Glutathione

## Abstract

Radiation therapy for head and neck cancer often causes xerostomia, a chronic salivary gland (SG) dysfunction driven by excessive reactive oxygen species (ROS), severely impacting the quality of life of patients. Existing treatments, such as artificial saliva or SG stimulants, offer only temporary relief. We developed a novel therapeutic system combining glutathione (GSH)-conjugated gelatin (Gel) hybrid protein-based cell carrier (GC) with adipose-derived mesenchymal stem cell (AdMSC) spheroids. This hybrid protein carrier (GC) extended the antioxidant activity of GSH by overcoming its short half-life and enabled efficient encapsulation and delivery of AdMSC spheroids to irradiation (IR)-damaged SGs. The 3D spheroids enhanced vascular endothelial growth factor (VEGF) expression through hypoxic core formation, promoting angiogenesis. In an IR-damaged mouse model, spheroid-encapsulated GC increased SOD2 expression and decreased 8-OHdG and NOX4 levels, effectively mitigating oxidative stress, fibrosis, and apoptosis, while accelerating angiogenesis. SG structural regeneration and functional recovery were confirmed using histological and immunohistochemical analyses following spheroid-encapsulating GC treatment. These results demonstrate the synergistic effects of ROS-scavenging activity from GC and paracrine signaling from AdMSC spheroids. This groundbreaking spheroid-encapsulating GC improves SG regeneration and functional recovery by integrating antioxidant activity with angiogenic effects, offering a paradigm-shifting solution for other oxidative stress-mediated tissue disorders.

## Highlights

• GSH-gelatin hybrid protein exerts prolonged antioxidant activity.
• GC can stably encapsulate and deliver AdMSC spheroids.
• AdMSC spheroids boost SG regeneration via hypoxia-induced angiogenesis.
• AdMSC spheroid encapsulating GC reduces oxidative stress, fibrosis, and apoptosis in IR-damaged SG.
• AdMSC spheroid encapsulating GC accelerate SG tissue regeneration and functional recovery.

## Graphical abstract

**Figure:**
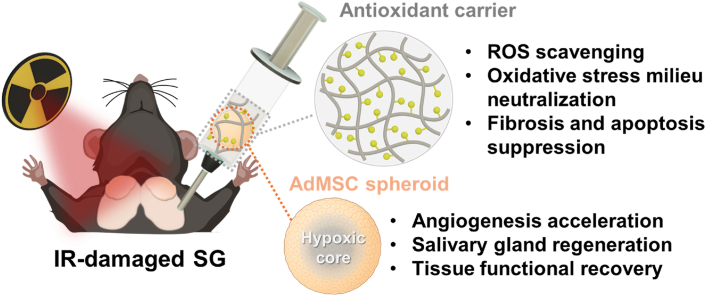

## 1 Introduction

Irradiation (IR) therapy is a standard treatment for head and neck cancer, but it often causes severe side effects, including xerostomia, a debilitating salivary gland (SG) disorder [[1], [2], [3]]. SGs are especially vulnerable to IR-induced damage, primarily due to the generation of reactive oxygen species (ROS), which continuously damage glandular tissues and impair function [4,5]. This reduction in saliva production compromises digestion, speech, antimicrobial defense, and electrolyte balance [3,6]. Consequently, xerostomia significantly diminishes the quality of life of patients, causing chronic discomfort and nutritional issues [7]. Current clinical treatments include (1) artificial saliva, which offers only transient and insufficient relief, and (2) SG stimulants, such as pilocarpine, which can cause various parasympathomimetic side effects [8,9]. Therefore, a fundamental therapeutic strategy capable of regenerating IR-damaged SG tissues and restoring their function is urgently needed.

Recent studies have explored the potential of adipose-derived mesenchymal stem cells (AdMSCs) for regenerating IR-damaged tissues. AdMSCs possess strong differentiation potential, IR resistance, and paracrine secretion abilities [10,11]. However, conventional 2D-cultured AdMSCs show limited regenerative efficacy due to insufficient cell-to-cell interactions, which are essential for proper differentiation and complex intercellular communication [12]. To overcome these limitations, 3D spheroid culture has emerged as a promising strategy to enhance AdMSC therapeutic efficacy. This method closely mimics native tissue architecture, supporting stemness and improving cellular resilience under stress conditions [13]. Notably, spheroids display significantly greater paracrine activity than their 2D counterparts, with prolonged and elevated secretion of key regenerative cytokines that promote tissue repair and modulate the inflammatory microenvironment. This enhanced secretory profile plays a pivotal role in facilitating angiogenesis and mitigating radiation-induced tissue damage [14]. Building on this approach, previous research has demonstrated that 3D AdMSC spheroids generate a hypoxic core, leading to the accumulation of hypoxia-inducible factor-1α (HIF-1α), which upregulates pro-angiogenic factors, such as VEGF [15]. Our group has successfully applied AdMSC spheroids in SG and laryngeal tissue regeneration, demonstrating their broad therapeutic potential [16,17]. Despite these promising results, challenges remain in delivering spheroids effectively to IR-damaged SGs. The conventional method, injection of spheroids suspended in Matrigel, hyaluronic acid, or similar carriers, often results in poor retention at the injury site, thereby reducing therapeutic efficacy [18]. To address this, various hydrogel carrier systems have been explored to improve spheroid retention. However, these carriers often lack intrinsic bioactive properties essential for promoting SG regeneration, limiting their overall therapeutic impact [19]. In particular, the ability to effectively scavenge reactive oxygen species (ROS) generated after IR treatment limits their overall therapeutic potential. Ionizing radiation interacts with intracellular water to produce excessive ROS, such as hydroxyl radicals (· OH), which trigger the secretion of pro-inflammatory cytokines (e.g., TNF-α, IL-6), promoting SG fibrosis and vascular damage [20]. This cascade compromises blood supply and tissue regeneration, ultimately reducing SG function. Furthermore, ROS downregulate antioxidant enzymes (e.g., SOD2, CAT), disrupting redox homeostasis and perpetuating chronic inflammation [21]. Therefore, there remains a need for developing an antioxidant cell carrier that can both encapsulate and deliver AdMSC spheroids to irradiated SGs and simultaneously neutralize early ROS surges, thereby mitigating oxidative stress, suppressing fibrosis, and supporting tissue regeneration.

We synthesized an antioxidant a glutathione-gelatin (GG) hybrid protein by functionalizing GSH onto gelatin, a structural protein. GSH, a tripeptide antioxidant composed of γ-glutamyl-cysteinyl-glycine, relies on the thiol group of cysteine for its powerful ROS-scavenging activity. It neutralizes ROS through three main mechanisms: (1) direct scavenging, (2) enzymatic reduction *via* glutathione peroxidase (GPx), and (3) redox cycling mediated by glutathione reductase (GR) [[22], [23], [24], [25]]. Despite these versatile antioxidant mechanisms, GSH has rarely been explored as a component within hydrogels or as a polymer-conjugated additive. Yu et al. developed a gelatin-gum arabic coacervate to encapsulate GSH, demonstrating thermal stability; however, their study did not address the short *in vivo* half-life or propose clinically viable strategies [26]. Similarly, Li et al. functionalized GSH onto chitosan and evaluated the antioxidant effects in cardiomyocytes, but lacked *in vivo* validation and long-term antioxidant assessment [27]. Therefore, validating sustained antioxidant activity of GSH-functionalized polymers during tissue regeneration is essential to overcome the short *in vivo* half-life of GSH (approximately 2–20 min in plasma) and holds significant value for tissue engineering applications [28].

Our GG hybrid protein exhibits intrinsic antioxidant activity and enables temperature-responsive behavior through gelatin's upper critical solution temperature (UCST) near 37 °C [29,30]. Additionally, tyrosine (Tyr) residues within gelatin can form di-tyrosine (Di-Tyr) bonds under specific conditions, establishing a robust interchain polymer network. Leveraging these properties, we developed an antioxidant cell carrier capable of spheroid encapsulation. Di-Tyr crosslinking was initiated through electron transfer in a ruthenium (Ru)/sodium persulfate (SPS) photocatalyst system under biocompatible blue light (450 nm) [31]. Compared to conventional UV-based crosslinking, this Ru/SPS + blue light system minimizes DNA double-strand breaks and cytotoxicity, enabling fast, efficient, and biocompatible gelation without the need for non-degradable methacrylate groups [32]. Furthermore, the system allows for easy incorporation of therapeutic agents, such as growth factors or stem cell spheroids, into the hydrogel precursor, which can then be molded into clinically relevant forms. Accordingly, we combined this photo-crosslinking approach with the GG hybrid protein to develop a novel antioxidant GG-based cell carrier (GC) that integrates antioxidant functionality with robust spheroid encapsulation.

To evaluate the preclinical efficacy of this system in restoring IR-damaged SG function, we conducted histological assessments (H&E for tissue architecture; Masson's Trichrome (MT) for fibrosis; and AB for acinar structures), along with immunohistochemical analyses (AQP5 as a water channel marker, Mist1 for acinar cells, and CD31 for angiogenesis). Additionally, we investigated antioxidant enzyme expression (SOD2), oxidative stress markers (8-OHdG, NOX4), and an apoptosis marker (cleaved caspase-3) to determine whether therapeutic outcomes were linked to ROS mitigation. The GC system exhibited strong antioxidant activity, reduced fibrosis, and inhibited apoptosis. Furthermore, the encapsulated spheroids promoted angiogenesis through VEGF secretion, contributing to SG tissue regeneration and functional recovery. Collectively, this integrated approach represents a new therapeutic paradigm for treating IR-induced xerostomia by uniting sustained ROS scavenging with spheroids-mediated tissue repair, ultimately improving patient outcomes and quality of life.

## 2 Results

### 2.1 Development of antioxidant hybrid protein

**Fig. 1:**
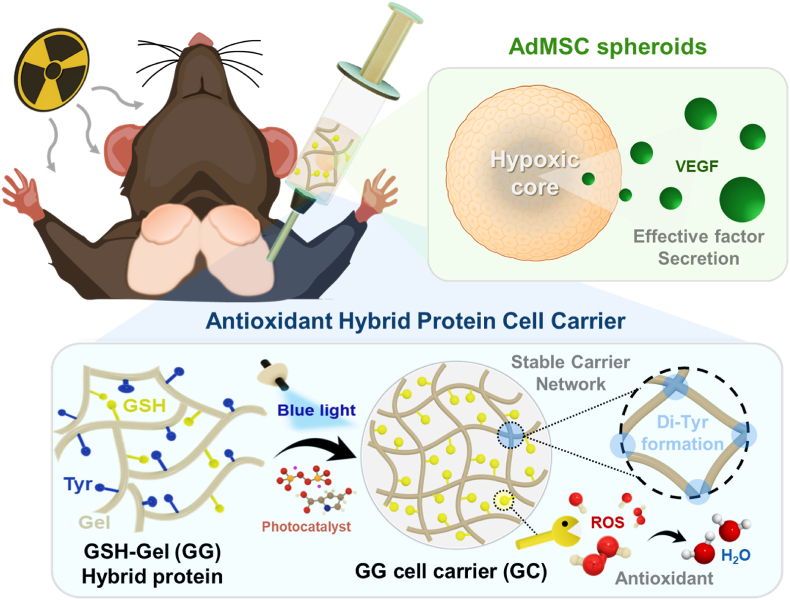
Schematic illustration of the AdMSC spheroid-encapsulating antioxidant GG carrier system for treating IR-damaged salivary glands.

In this study, we propose an innovative spheroid-encapsulating GC system to address IR-induced xerostomia (Fig. 1). This system consisted of AdMSC spheroids embedded within a GC that exhibited ROS-scavenging capability. The GC effectively suppressed oxidative stress following IR exposure and delivered spheroids to the damaged site, where the spheroids promoted angiogenesis and tissue regeneration through VEGF secretion, thereby maximizing therapeutic outcomes.

**Fig. 2:**
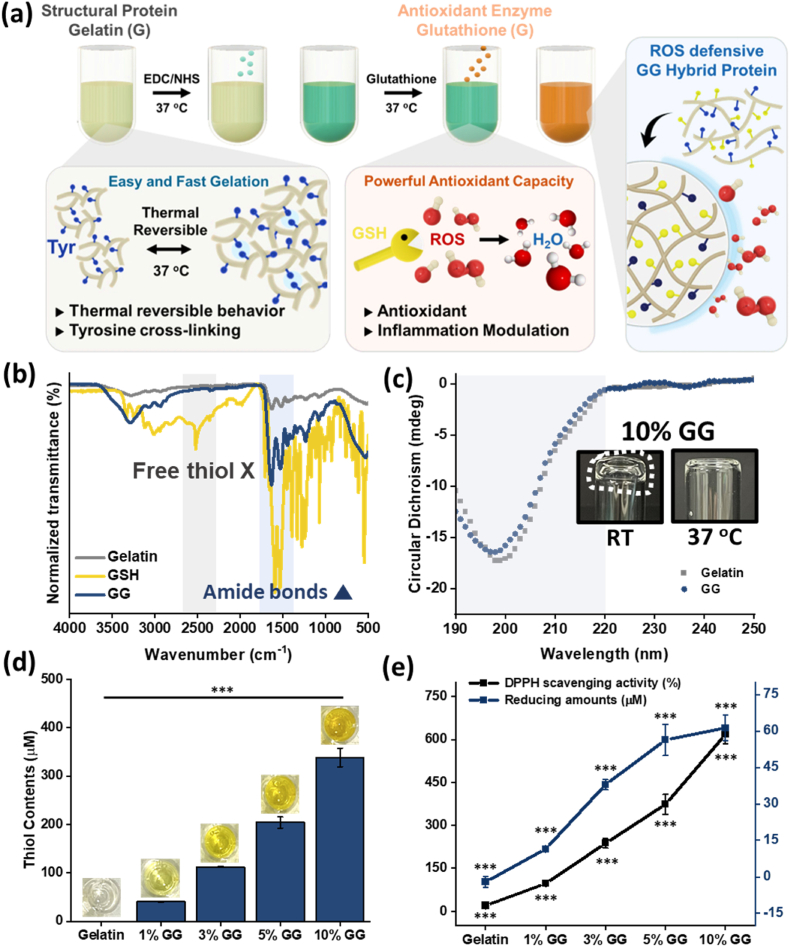
**Development of antioxidant glutathione-gelatin (GG) hybrid protein.** (a) Schematic illustration of the synthesis of GG *via* EDC/NHS coupling and subsequent blue-light-mediated crosslinking to form a stable antioxidant GG network. (b) Fourier-transform infrared (FT-IR) spectra of gelatin (gray line), GSH (yellow line), and GG (deep blue line). (c) Circular dichroism (CD) analysis confirming the preservation of gelatin's secondary structure post-conjugation and optical images showing thermo-reversible gelation of 10% (w/v) GG at 25 °C and 37 °C. (d) Quantification of thiol group content (μM ) in 1%, 3%, 5%, and 10% (w/v) GG. (e) Antioxidant properties of GG assessed using a 2,2-diphenyl-1-picrylhydrazyl (DPPH) radical scavenging assay (black line) and total antioxidant capacity assay (deep blue line), showing a concentration-dependent increase in activity. Data are shown as mean ± SD (n = 5). Statistical analysis was performed using one-way ANOVA followed by Tukey's post-hoc test. ^ns^*P* > 0.05, ∗*P*≤ 0.05, ∗∗*P*≤ 0.01, and ∗∗∗*P*≤ 0.001.

To develop the antioxidant hybrid protein, gelatin, a structural protein derived from collagen, was selected as the backbone matrix. GSH, a natural antioxidant synthesized in the liver, was conjugated to the gelatin matrix *via* 1-ethyl-3-(3-dimethylaminopropyl) carbodiimide/N-hydroxysuccinimide (EDC/NHS) coupling chemistry. Fig. 2a illustrates the synthesis process of the GG, which demonstrated potent antioxidant activity and supported both thermal and covalent bonding-based gelation.

To confirm successful amide bond formation between GSH and gelatin, Fourier-transform infrared (FT-IR) spectroscopy was conducted (Fig. 2b). Gelatin (gray line) showed characteristic peaks at 1650 cm^−1^ (Amide I) and 1560 cm^−1^ (Amide II). The NH stretching and thiol peaks of GSH (yellow line) were detected at 3000–3500 cm^−1^ and 2500 cm^−1^, respectively. The carboxyl groups of gelatin and the primary amine groups of GSH formed amide bonds *via* EDC/NHS coupling to synthesize GG. Notably, the amide II bonds of GG (deep blue line) exhibited higher transmittance than gelatin. These results confirm the successful synthesis of GG. The absence of significant thiol peaks of GSH in the GG was due to the low content of detectable thiol groups within the macromolecular gelatin matrix, in contrast with the free thiols present in GSH.

Circular dichroism (CD) analysis was conducted to evaluate the potential denaturation of gelatin's secondary structure following EDC/NHS coupling with GSH. As shown in Fig. 2c, the intrinsic random coil structures (≤200 nm) of both gelatin and GG were well preserved. Moreover, the UCST behavior, a unique property of gelatin linked to its gelation capability, was also retained in GG, as confirmed by the optical images.

The thiol groups, which are introduced by GSH and present in GG, were quantified using a thiol assay (Fig. 2d). The results indicated that gelatin, 1%, 3%, 5%, and 10% (w/v) GG contained −9.56 × 10^−6^ (± 7.76× 10^−7^)μM, 40.37 (±0.95) μM, 112.14 (±0.87) μM, 203.83 (±11.58) μM, and 338.4 (±19.05) μM of thiol groups, respectively. Therefore, the successful synthesis of GG was quantitatively confirmed. These thiol groups in GG are important functional groups that provide antioxidant activity, thus alleviating oxidative stress and tissue fibrosis. In Fig. 2e, to evaluate the antioxidant capacity of GG, a DPPH radical scavenging assay (black line) and a total antioxidant capacity test (deep blue line) were conducted. As the w/v% of GG increased, the thiol group content also increased, which in turn enhanced antioxidant activity, with 10% GG achieving approximately 70% ROS scavenging compared to no activity in gelatin.

The FT-IR analysis, thiol quantification, and antioxidant assays collectively confirmed the successful synthesis of GG. Furthermore, these systematic analyses verified that the secondary structural characteristics of gelatin were preserved, while the antioxidant activity of GSH was effectively integrated into the gelatin matrix, supporting its potential to suppress oxidative stress and mitigate tissue fibrosis.

### 2.2 Fabrication of GC and its characterization

**Fig. 3:**
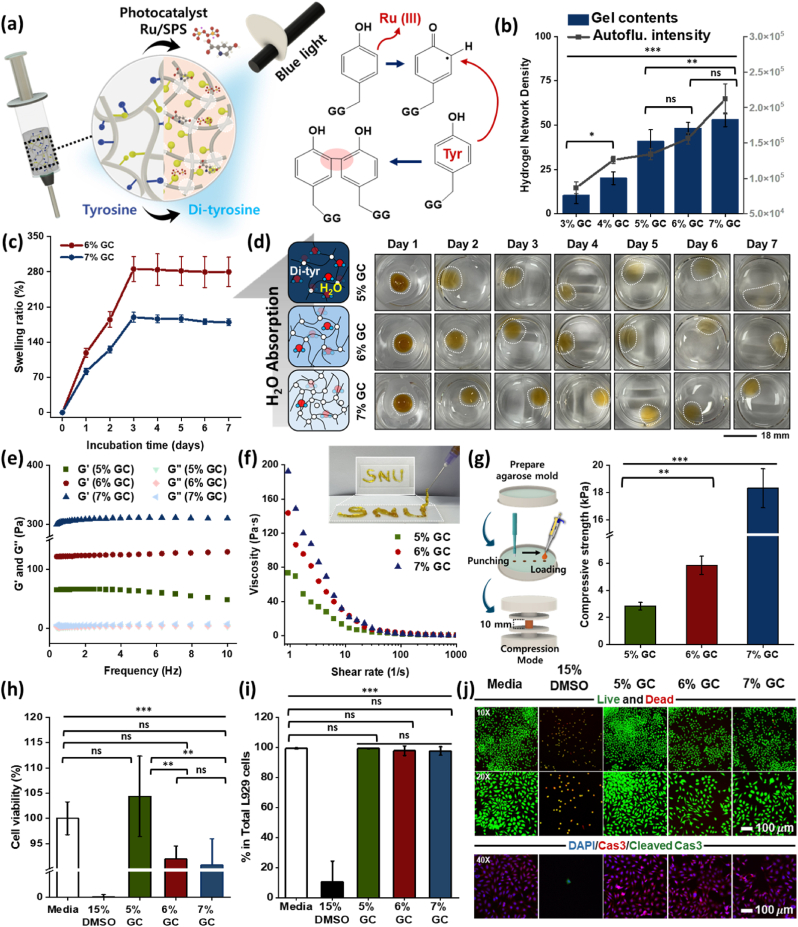
**Biocompatible blue light-induced fabrication of antioxidant GC.** (a) Schematic illustration of antioxidant GG cell carrier (GC) and chemical mechanism of blue light-induced gelation. (b) Assessment of GC network density based on Gel content (%, deep blue bars) and the autofluorescence intensity of Di-Tyr (a.u., gray line). (c) Swelling ratio (%) of 6% and 7% (w/v) GCs over time. (d) Optical images of 5–7% (w/v) GCs showing physical swelling behavior over 7 d. (e) Elastic modulus (G′) and loss modulus (G’’) of 5–7% (w/v) GCs. (f) Shear-thinning behavior and injectability of 5-7% (w/v) GCs. (g) Schematic and quantification of ultimate compressive strength (kPa) from compression testing of 5–7% (w/v) GCs. (h) Biocompatibility evaluation of 5–7% (w/v) GCs using L929 cells, assessed using a cell viability assay. (i) Flow cytometric quantification of cell viability. (j) Confocal images of Live/Dead (green/red) and DAPI/Caspase-3/Cleaved Caspase-3 (blue/green/red)-stained L929 cells cultured in GC extracts after 3 d. Data are shown as mean ± SD (n = 5). Statistical analysis was performed using one-way ANOVA followed by Tukey's post-hoc test. ^ns^*P* > 0.05, ∗*P*≤ 0.05, ∗∗*P*≤ 0.01, and ∗∗∗*P*≤ 0.001.

After confirming successful GG synthesis, a biocompatible photo-crosslinking technique was applied to fabricate GC. Upon exposure to dental LED blue light (450 nm), the photocatalyst (ruthenium [Ru] and sodium persulfate [SPS]) facilitated electron transfer from the Tyr groups of GG. Ru was photolyzed in the presence of SPS to generate sulfate radicals that oxidize Tyr residues. These oxidized Tyr residues interacted with nearby Tyr residues, forming covalent di-tyrosine (Di-Tyr) bonds, which enabled the formation of a structurally stable and biocompatible cell carrier (Fig. 3a).

The physical, mechanical, and rheological properties of GC were affected by GG concentration. First, the network density was assessed *via* Gel content (deep blue bar) and the autofluorescence intensity of Di-Tyr (gray line), indicating enhanced structural stability with increasing GG concentration (Fig. 3b). This stability is critical for spheroid encapsulation within the GC, aimed at SG regeneration.

In the subsequent experiments, only 5–7% (w/v) GCs were used because 3% and 4% (w/v) GCs, although biocompatible (Fig. S1), had inadequate physical properties, rendering them unsuitable as cell carriers (Fig. S2 and S3). Conversely, higher concentration of 8-10% (w/v) GCs were also excluded despite their high mechanical stiffness (Fig. S4a). These formulations exhibited excessively high initial viscosities (Fig. S4b), which led to significant aggregation and needle clogging during the injection process (21G needle; Fig. S4c). Such rheological limitations not only hinder precise, minimally invasive delivery to the SG but could also impose detrimental shear stress on the encapsulated AdMSC spheroids. Therefore, 5-7% (w/v) was identified as the optimal concentration range to balance structural integrity with reliable clinical injectability.

As shown in Fig. 3c, the swelling ratios of 6% and 7% (w/v) GCs were 240% and 160%, respectively, and both reached swelling equilibrium by day 3. The 5% (w/v) GC maintained its structure for 7 d; however, due to its limited structural stability, weight measurements were not possible. Therefore, the swelling behavior of 5% GC was presented qualitatively (Fig. 3d).

Rheological evaluations were conducted to assess structural stability (Fig. 3e). The storage modulus (G′) and loss modulus (G’’) of GC were as follows: 5% (w/v) GC (G’ = 65.82 ± 3.97 Pa, G’’ = 7.86 ± 0.62 Pa), 6% (w/v) GC (G’ = 126.2 ± 16.62 Pa, G’’ = 4.01 ± 0.21 Pa), and 7% (w/v) GC (G’ = 315.36 ± 6.54 Pa, G’’ = 60.89 ± 0.57 Pa). The increase in storage modulus with GC concentration, alongside the consistently lower loss modulus, suggests enhanced Di-Tyr formation, contributing to improved GC's elasticity and structural stability for spheroid support. Notably, the time-dependent rheological analysis over 7 days revealed a gradual decrease in G′ values for all GCs, which can be attributed to the progressive structural relaxation and partial degradation of the gelatin backbone in an aqueous environment (Fig. S5). Specifically, the G′ of the 7% (w/v) GC changed from 211.64 Pa to 83.70 Pa by day 7. Despite this reduction, all GC groups retained sufficient mechanical integrity to support the spheroid without complete disintegration, demonstrating a controlled degradation profile suitable for long-term tissue regeneration.

Furthermore, all GC formulations exhibited distinct non-Newtonian shear-thinning behavior (Fig. 3f), with viscosity decreasing significantly as the shear rate increased from 1 to 1000 s^−1^. Specifically, the initial viscosity at a low shear rate (1 s^−1^) increased with concentration: 73.48 Pa s (5% w/v), 143.6 Pa s (6% w/v), and 192.45 Pa s (7% w/v). This behavior is visually validated by the successful injectability and high shape fidelity of the GC, as demonstrated by precisely the hand-written “SNU” logo. These results indicate that GC possesses the ideal rheological properties for the effective and minimally invasive delivery of spheroids to IR-damaged SG.

As another index of structural stability, the mechanical properties of 5–7% (w/v) GCs were evaluated via compressive strength testing (Fig. 3g and S6). Compressive strength increased with GC concentration: 2.836, 5.862, and 18.318 kPa for 5%, 6%, and 7% (w/v) GC, respectively. This trend can be attributed to improved structural stability resulting from enhanced network density as GG concentration increased.

Biocompatibility of GCs at different concentrations was evaluated according to ISO 10993 standards using L929 cells (Fig. 3h). On day 3, 5–7% (w/v) GCs exhibited cell viabilities exceeding 70%, surpassing the cytotoxicity grade 1 threshold. To further validate the biocompatibility of the GC, we performed a quantitative flow cytometric analysis using Zombie Aqua staining. As shown in Fig. 3i and S7, the percentage of live L929 cells remained remarkably high across all GC groups, with 5-7% (w/v) GCs showing live cell populations of 99.4%, 97.98%, and 97.72%, respectively. These values were comparable to the healthy Media group (99.34%) and significantly higher than the 15% DMSO group (10.74%), which exhibited massive cell death. Additionally, the biocompatibility was visually confirmed through Live/Dead and immunofluorescence staining after 3 days of culture in GC extracts (Fig. 3j). The Live/Dead assay revealed a predominant population of green-fluorescent (live) cells with characteristic elongated morphology and active stretching, further supporting the CCk-8 and flow cytometry data. More importantly, considering the potential DNA damage or stress caused by GC extracts, we analyzed the expression of cleaved caspase-3, a hallmark of programmed cell death. As demonstrated in the DAPI/caspase-3/cleaved caspase-3 merged images, the GC groups showed minimal to no signals for cleaved caspase-3, similar to the Media group, whereas the 15% DMSO groups displayed prominent apoptotic signaling. Also, the expression of cleaved caspase-3 was quantitatively analyzed using flow cytometry (Fig. S8). The relative mean fluorescence intensity (MFI) was comparable to those of the Media group, with no significant differences observed. Specifically, while the 15% DMSO group showed a dramatic increase in the apoptotic populations (3.47), the GC maintained minimal levels (approximately 1), consistent with the results from the qualitative immunofluorescence analysis. As a result, 5–7% (w/v) GCs were confirmed to be biocompatible for spheroid encapsulation, with 5% (w/v) GC demonstrating the highest cell viability.

The physical properties of GCs were tunable based on GG concentration. Furthermore, the photo-crosslinking fabrication process, involving minimal steps with Ru/SPS and blue light, was biocompatible and operationally simple, offering significant advantages for spheroid encapsulation and potential clinical translation in SG regeneration.

### 2.3 Antioxidant activity of GC

**Fig. 4:**
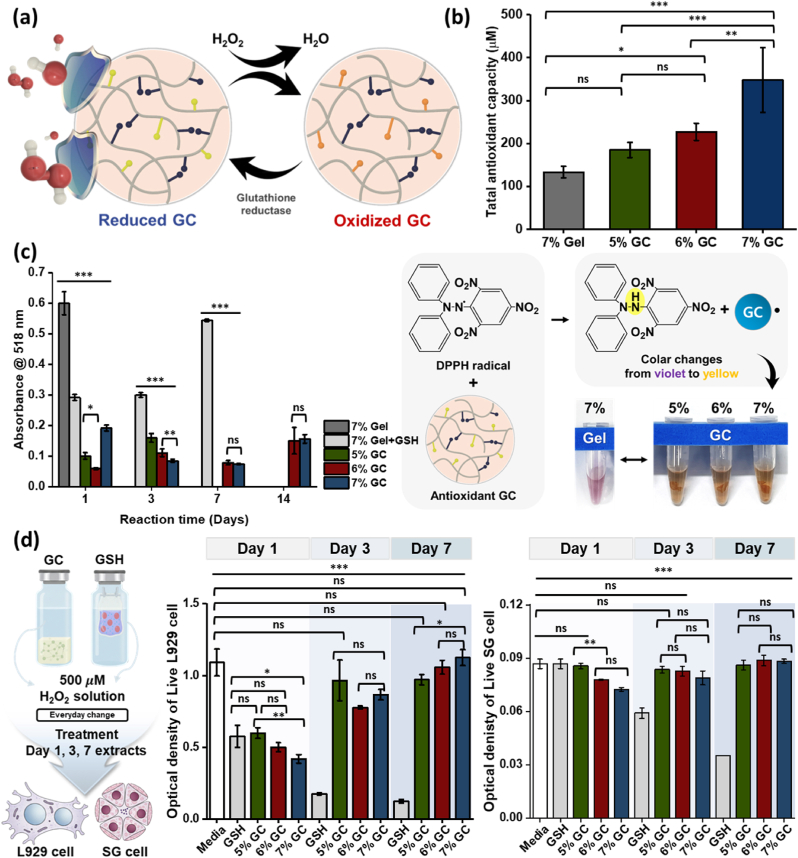
**Sustained antioxidant activity of GC**. (a) Representative illustration of the antioxidant mechanism of GC. (b) Total antioxidant capacity of 7% (w/v) gelatin and 5–7% (w/v) GCs. (c) Long-term antioxidant activity measured *via* DPPH radical scavenging in 7% (w/v) gelatin, 40 mM GSH-loaded 7% (w/v) gelatin, and 5–7% (w/v) GCs over 14 d. (d) Overcoming the short half-life of GSH for sustained *in vitro* antioxidant efficacy of GC. Data are presented as mean ± SD (n = 5). Statistical analysis was performed using one-way ANOVA followed by Tukey's post-hoc test. ^ns^*P* > 0.05, ∗*P*≤ 0.05, ∗∗*P*≤ 0.01, and ∗∗∗*P*≤ 0.001.

To evaluate the antioxidant capacity of the GC, a critical component of the SG regenerative system for mitigating oxidative stress in IR-damaged SG, we investigated its ROS scavenging properties (Fig. 4). The antioxidant functionality of GC is driven by thiol groups from GG, which directly neutralize ROS in the absence of enzymatic cofactors, such as GPx and GR. In this environment, the thiols react with ROS, such as H_2_O_2_, to form disulfides (oxidized glutathione; glutathione disulfide, GSSG) and water (2GSH + H_2_O_2_ → GSSG + 2H_2_O), effectively reducing oxidative stress. This non-enzymatic mechanism ensures sustained ROS scavenging, maintaining the ROS-scavenging capacity of GC. Fig. 4a represents the antioxidant response of GC in oxidative-stressed SG.

The antioxidant activity of GC was quantitatively evaluated using a total antioxidant capacity assay (Fig. 4b). Compared to the 7% (w/v) gelatin hydrogel (Gel), which exhibited minimal antioxidant capacity, with an absorbance of approximately 0.21 (± 0.004) a.u. (gray bar), GC demonstrated concentration-dependent antioxidant activity, with 5%, 6%, and 7% (w/v) GCs showing absorbance values of 0.26 (± 0.044), 0.29 (± 0.027), and 0.37 (± 0.046) a.u., respectively. These results highlight the enhanced antioxidant potential of GC, attributable to the chemically integrated thiol groups from GSH within GG, which were absent in Gel, thereby contributing to ROS neutralization and protection against tissue fibrosis.

The DPPH radical scavenging activity of GC was assessed over 14 d under a sustained ROS environment to evaluate its long-term antioxidant activity, a critical aspect of the SG regenerative system for mitigating oxidative stress in IR-damaged SG (Fig. 4c). To ensure consistent experimental conditions and prevent interference from the photocatalyst (Ru/SPS) color during absorbance measurements, all samples were dialyzed with DI water every 2 d to remove residual photocatalyst. Each sample was then immersed in DPPH reagent, and the supernatant was analyzed at each time point. The DPPH reagent was replaced to maintain a continuous oxidative environment. On day 1, 7% (w/v) Gel (dark gray) exhibited a significantly higher absorbance (0.6 ± 0.037 a.u. at 518 nm) than the other groups, indicating negligible antioxidant activity, since no color change (violet to yellow) occurred due to the absence of ROS scavenging capacity. In contrast, physically 40 mM GSH-encapsulating 7% (w/v) Gel (light gray) displayed an absorbance of 0.29 ± 0.01 a.u., reflecting partial antioxidant activity. However, this antioxidant activity was sustained only until day 3, after which it diminished, likely due to the rapid oxidative depletion of the physically encapsulated GSH in the ROS-rich environment, as the Gel matrix failed to stabilize GSH, resulting in low stability and reduced effectiveness. The 5% (w/v) GC exhibited antioxidant activity; however, its weak mechanical properties prevented reliable supernatant collection, rendering further measurements challenging by day 14. Meanwhile, 6% and 7% (w/v) GCs, with initial absorbance values of 0.06 (± 0.003) a.u. and 0.19 (± 0.01) a.u., respectively, maintained structural integrity and exhibited sustained antioxidant activity over 14 d. Their absorbances increased to 0.15 (± 0.044) a.u. and remained at 0.16 (± 0.014) a.u., respectively. These results, visually supported by accompanying illustration, confirm the strong long-term antioxidant capacity of 6% and 7% (w/v) GCs, driven by chemically conjugated GSH, making them highly suitable for protecting SGs from oxidative stress and promoting tissue regeneration following IR therapy.

Furthermore, the cytoprotective efficacy of GC under chronic oxidative stress was evaluated to determine its potential to overcome the short half-life of free GSH (Fig. 4d). In clinical scenarios, sustained protection against persistent ROS is essential; however, free GSH often loses its bioactivity rapidly due to oxidative depletion or degradation. To simulate this environment, GCs and free GSH solutions were exposed to 500 μ M H_2_O_2_ solution, which was refreshed daily to provide a constant oxidative challenge. Extracts collected at pre-determined intervals (Days 1, 3, and 7) were then treated onto L929 fibroblasts and SG cells to assess their remaining protective capacity. The comparative analysis revealed that free GSH rapidly lost its cytoprotective capacity, with L929 and SG cell viability dropping significantly by Day 3 and reaching negligible levels by Day 7 under daily refreshed oxidative stress. In stark contrast, all GC groups, particularly the 7% (w/v) GC, demonstrated robust and sustained antioxidant efficacy throughout the 7-day period. L929 cells treated with 7% (w/v) GC extracts maintained a high optical density (approximately 1.125 at day 7), which was significantly higher than the free GSH group. Similar trends were observed in SG cells, where GC formulations effectively neutralized persistent H_2_O_2_ exposure, preserving cell survival even at the latest time point. These results collectively indicate that the chemical conjugation of GSH within the gelatin backbone prevents its rapid oxidative depletion, providing steric protection to the thiol groups, thereby enhancing their chemical stability compared to the unbound, freely mobile state of GSH. Plus, the hydrogel matrix functions as a localized antioxidant reservoir, restricting the rapid diffusion and subsequent degradation that typically limit the biological half-life of conventional antioxidants. Consequently, this stable integration effectively overcomes the intrinsic half-life limitations of GSH, ensuring prolonged therapeutic efficacy in persistent ROS-rich environments.

**Fig. 5:**
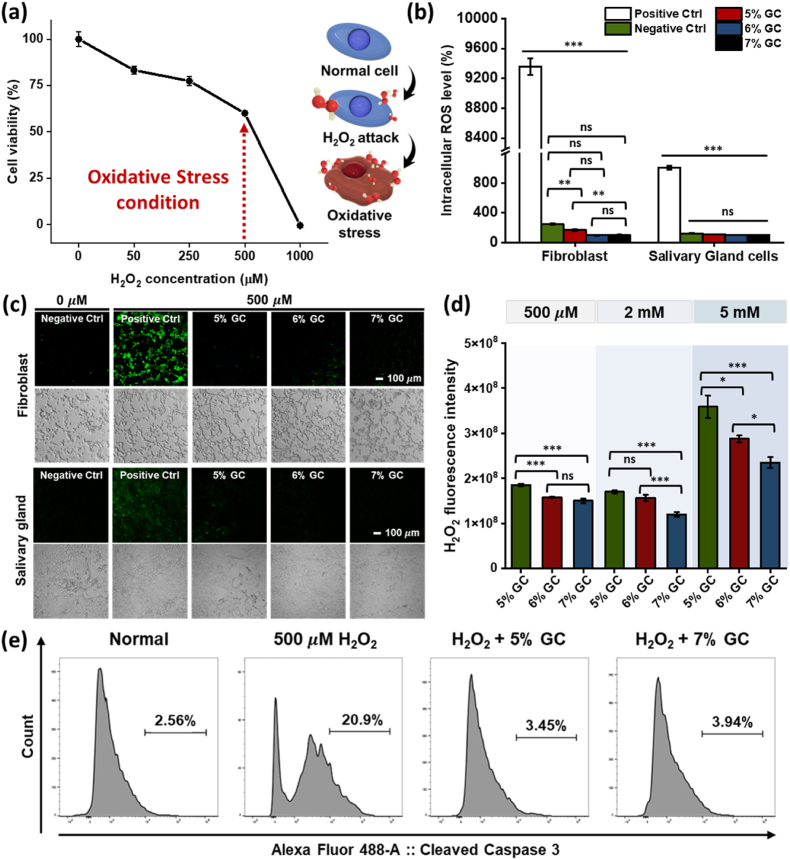
**Intracellular antioxidant capacity of GC.** (a) Optimization of the oxidative stress environment based on NIH3T3 cell viability (%) following treatment with 0–1000 μM H_2_O_2_. (b) Intracellular ROS levels (%) in NIH3T3 cells and salivary gland cells under the following conditions: normal cells (negative control, NC), cells treated with 500 μM H_2_O_2_ (positive control, PC), and cells exposed to supernatants from 5% to 7% (w/v) GCs following 500 μM H_2_O_2_ stimulation. (c) Fluorescence confocal images of intracellular ROS probe-stained NC, PC, and 5-7% (w/v) GCs. (d) Evaluations of H_2_O_2_ scavenging intensity of 5-7% (w/v) GCs at various H_2_O_2_ concentrations (500 μ M, 2 mM, and 5 mM) (n = 3). (e) Representative histograms and quantitative percentages of Cleaved Caspase-3 expressing salivary gland cells (n = 3). Data are presented as mean ± SD (n = 5). Statistical analysis was performed using one-way ANOVA followed by Tukey's post-hoc test. ^ns^*P* > 0.05, ∗*P*≤ 0.05, ∗∗*P*≤ 0.01, and ∗∗∗*P*≤ 0.001.

To confirm the *in vitro* antioxidant activity of GC, its capacity to mitigate oxidative stress in IR-damaged SG was evaluated using an intracellular ROS level assay. As shown in Fig. 5a, the concentration of H_2_O_2_ used to induce oxidative stress was optimized to avoid apoptosis. A concentration of 500 μ M H_2_O_2_ was determined to be optimal, since it induced oxidative stress while maintaining cell viability. Normal NIH3T3 cells served as a negative control (NC), with intracellular ROS levels normalized to 100%. In contrast, the positive control (PC), NIH3T3 cells exposed to 500 μ M H_2_O_2_, exhibited a ROS level of 373.94% relative to that of the NC. When 5–7% (w/v) GCs were pre-treated with 500 μ M H_2_O_2_ and their supernatants subsequently applied to NIH3T3 cells, intracellular ROS levels were significantly decreased: 5%, 6%, and 7% (w/v) GCs resulted in ROS levels of 79.18 (± 6.75%), 69.83 (± 6.234%), and 59.45 (± 2.877%), respectively (Fig. 5b). These results demonstrate the concentration-dependent ROS scavenging capacity of GC, attributable to the conjugated GSH, which effectively neutralizes H_2_O_2_ *via* thiol-mediated antioxidant activity, thereby mitigating oxidative stress.

To further validate the ROS protective effect of GC in a disease-relevant cell type, SG cells were applied to the same oxidative stress experiments. As shown in Fig. 5b, NC displayed 100 ± 8.76% of ROS levels, whereas PC elevated intracellular ROS to 9356.03± 110.09%. 5%-7% (w/v) GCs markedly reduced ROS levels to 248± 10.65%, 168± 10.82%, 99.83 ± 3.47%, respectively. These quantitative findings were corroborated by qualitative confocal microscopy images (Fig. 5c). In both 500 μ M H_2_O_2_ exposed NIH3T3 fibroblasts and SG cells, intense green fluorescence was observed in the PC, whereas 5-7% (w/v) GCs resulted in a reduced fluorescence intensity.

The robustness of GC's antioxidant defense was further challenged by evaluating its H_2_O_2_ scavenging intensity across a gradient of extreme oxidative conditions (Fig. 5d). Interestingly, at a relatively low concentration of H_2_O_2_ (500 μ M), 5-7% (w/v) GC exhibited high scavenging efficiency with no significant differences among the groups, as the antioxidant capacity of even the lowest GC concentration was sufficient to neutralize the limited ROS presence. However, as the oxidative challenge intensified (2 mM and 5 mM H_2_O_2_), the concentration-dependent antioxidant efficacy of the GCs became markedly pronounced. Under the most severe condition (5 mM H_2_O_2_), where the ROS load exceeded the capacity of lower concentration GCs, the 7% (w/v) GC displayed a significantly superior reduction in fluorescence intensity compared to the 5% (w/v) GC group. This divergence underscores the higher density of chemically conjugated thiol groups within the 7% (w/v) GC network provides a more formidable and reliable barrier against severe oxidative insults that may overwhelm conventional antioxidant systems.

The biological impact of this superior ROS neutralization was confirmed through flow cytometric analysis of cleaved caspase-3 (Fig. 5e). Exposure to 500 μ M H_2_O_2_ triggered a sharp increase in the pro-apoptotic cell populations (20.9%), reflecting severe oxidative damage. In contrast, pre-treatment with 5% and 7% (w/v) GCs effectively rescued the SG cells, drastically lowering the percentage of cleaved caspase-3 positive cells to 3.45% and 3.94%, respectively. These levels were nearly identical to the normal control (2.56%), highlighting that GC-mediated ROS scavenging directly suppresses the caspase-mediated apoptotic signaling cascade. Collectively, these results validate that GC acts as a bioactive cell carrier that preserves cellular integrity by actively interrupting the transition from oxidative stress to programmed cell death.

### 2.4 Formation and functional characterization of AdMSC spheroids and their integration with GCs

**Fig. 6:**
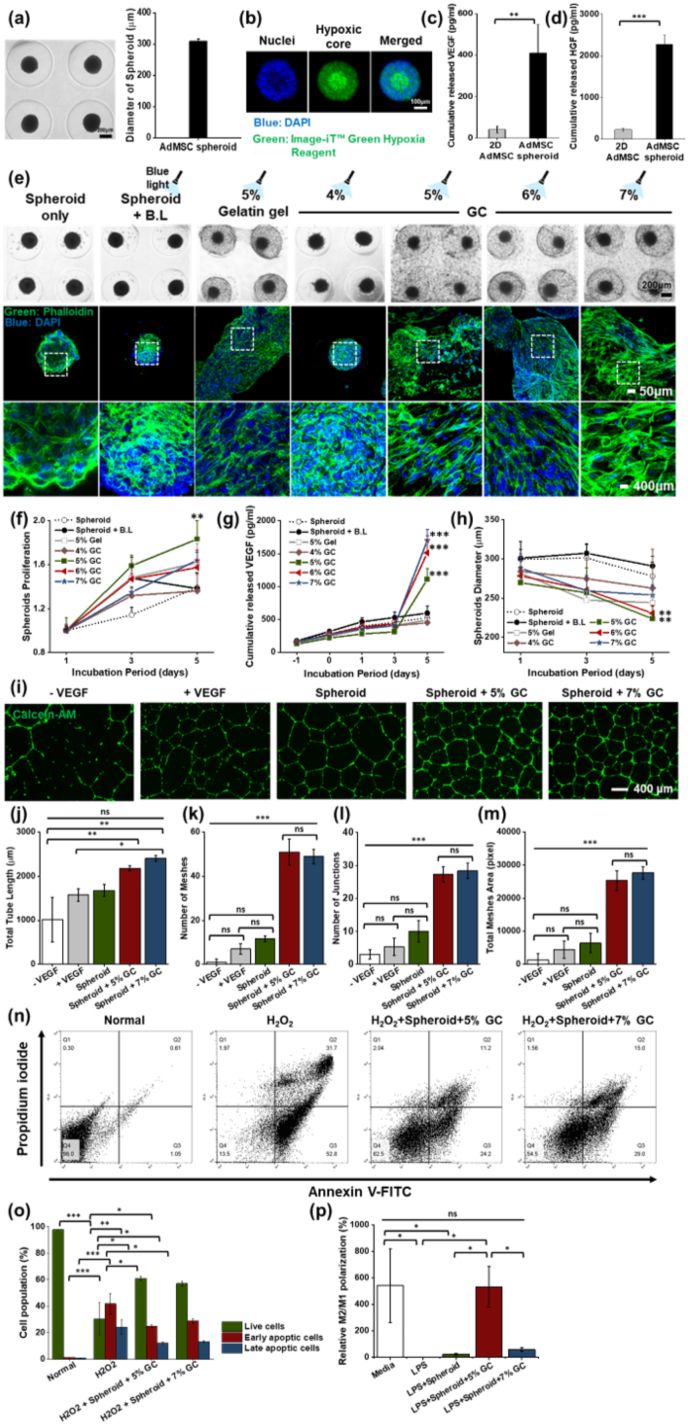
**Functional characterization of AdMSC spheroids and their integration with GC.** (a) Bright-field images and size distribution of spheroids generated in agarose molds targeting a diameter of 300 μm. (b) Representative confocal images of hypoxic core staining in spheroids, showing nuclei (blue) and hypoxic regions (green). (c, d) ELISA quantification of cumulative VEGF and HGF secretion from 2D-cultured AdMSCs and spheroids after 3 days culture. (e) Bright-field and confocal fluorescence images of spheroids alone, spheroids exposed to blue light (B.L), spheroids encapsulating 5% (w/v) gelatin hydrogel, or spheroids encapsulating 4–7% (w/v) GCs and photo-crosslinked under 450 nm blue light. Samples were stained with phalloidin (green, F-actin) and DAPI (blue, nuclei); dotted boxes indicate magnified regions. (f) Quantification of normalized cell proliferation within the GC in each condition over 1, 3, and 5 days incubation (n = 3). (g) Cumulative VEGF secretion from encapsulated spheroids measured at 1, 3, and 5 days (n = 5). (h) Spheroid diameter measurements over the incubation period, indicating the degree of spheroid spreading within each matrix (n = 10). (i) Representative HUVEC tube formation images after treatment with conditioned media from VEGF-free control (-VEGF), VEGF-supplemented media (+VEGF), spheroids alone, spheroid encapsulating 5% (w/v) GC, or spheroid encapsulating 7% (w/v) GC groups. (j–m) Quantitative tube formation parameters, including total tube length, number of meshes, number of junctions, and total mesh area, respectively (n = 4). (n) Representative Annexin V-FITC/propidium iodide flow cytometry plots of H_2_O_2_-treated SG cells in the absence or presence of spheroid encapsulating 5% (w/v) GC or spheroid encapsulating 7% (w/v) GCs. (o) Quantification of live, early apoptotic, and late apoptotic cell populations under each condition (n = 3). (p) Relative M2/M1 polarization of macrophages after LPS stimulation and treatment with conditioned media with spheroids and spheroids encapsulating GCs as determined by ELISA (n = 3). Data are presented as mean ± SD. Statistical analysis was performed using one-way ANOVA followed by Tukey's post-hoc test. ^ns^*P* > 0.05, ∗*P*≤ 0.05, ∗∗*P*≤ 0.01, and ∗∗∗*P*≤ 0.001.

AdMSC spheroids were first fabricated using agarose molds targeting a diameter of 300 μm, and after 3 days of culture they exhibited a mean diameter of approximately 300 μm, confirming a highly uniform size distribution suitable for downstream encapsulation (Fig. 6a). Even across 12 independently fabricated batches (n = 12), spheroid formation consistently yielded structures with comparable diameters, indicating robust reproducibility of the fabrication process. These data demonstrate that well-formed and size-controlled AdMSC spheroids were reproducibly obtained as a consistent 3D platform for subsequent functional assays. Hypoxic core staining of these spheroids revealed a pronounced hypoxic region in the central zone, indicating the formation of a hypoxia-like microenvironment under 3D culture conditions (Fig. 6b). Consistent with this notion, ELISA measurements demonstrated that cumulative VEGF and HGF secretion levels were significantly higher in AdMSC spheroids than in 2D-cultured AdMSCs, confirming that the 3D spheroid configuration confers a clear paracrine advantage at the protein level (Fig. 6c and d).

After encapsulation into 4–7% (w/v) GCs, spheroid morphology was monitored by bright-field imaging and phalloidin/DAPI staining, which showed progressive stretching, inter-spheroid connectivity, and cell outgrowth from the spheroid core into the surrounding matrix over a 5-day culture period, particularly in 5% and 7% (w/v) GCs (Fig. 6e and Fig. S9a). These observations indicate that AdMSC spheroids are highly compatible with the GC and can actively extend into the surrounding matrix rather than remaining as static aggregates, suggesting favorable behavior for *in vivo* engraftment. Quantitative analysis using the Alamar Blue assay revealed that cell proliferation within the GC was highest in 5% (w/v), followed by 5% (w/v) gelatin gel, whereas 4% (w/v) GC exhibited limited gel stability and suboptimal cell support (Fig. 6f). In parallel, cumulative VEGF secretion increased with GC stiffness, with 7% (w/v) GC showing the highest VEGF level followed by 6% and 5% (w/v) GCs, suggesting that a relatively denser matrix further augments angiogenic cytokine release while still preserving spheroid-mediated paracrine activity (Fig. 6g).

The degree of spheroid spreading within the GC matrix was assessed by measuring spheroid diameter over time, revealing a gradual reduction in diameter as cells radially migrated into the hydrogel, with the most pronounced decrease observed in 5% (w/v) GC (Fig. 6h, Fig. S9b and S9c). This diameter reduction reflects active outward migration and partial loosening of the spheroid structure, implying that 5% (w/v) GC provides a mechanically permissive environment that promotes matrix integration and broad spatial distribution of paracrine factors. Building on this, tube-formation assays using endothelial cells showed that conditioned media from the spheroid + GC groups, especially spheroid +5% (w/v) GC and spheroid + 7% (w/v) GC, markedly enhanced total tube length, mesh number, junction number, and mesh area compared with VEGF-negative control, VEGF-treated control, or spheroids alone, indicating that GC-supported spheroids exert a stronger pro-angiogenic paracrine effect than either component alone (Fig. 6i–m). Furthermore, under H_2_O_2_-induced oxidative stress, Annexin V/PI staining demonstrated that co-culture with spheroid + GC (5% or 7% w/v) significantly increased the proportion of live cells while reducing early and late apoptotic populations compared with H_2_O_2_ alone, highlighting that spheroid encapsulating GCs provide pronounced cytoprotective and anti-apoptotic effects at the cellular level (Fig. 6n and o). In addition, viability assessment of spheroids under H_2_O_2_ treatment revealed that the spheroids exhibit resistance to oxidative stress without significant cellular damage, indicating that they maintained their functional capacity within ROS-rich environment (Fig. S10). Building on these cytoprotective effects, we next investigated whether conditioned media derived from spheroid encapsulating GCs could also modulate macrophage polarization under inflammatory conditions. When the media and LPS-only groups were considered as controls, all treatments with conditioned media from AdMSC spheroids (LPS + spheroid), LPS + spheroid encapsulating 5% (w/v) GC, and LPS + spheroid encapsulating 7% (w/v) GC promoted a shift in macrophages from an M1 toward an M2 phenotype (Media 541%; LPS-only 0.12%; LPS + spheroid 22%; LPS + spheroid encapsulating 5% w/v GC 533%; LPS + spheroid encapsulating 7% w/v GC 59%). Among these, the media conditioned from spheroid encapsulating 5% (w/v) GC induced the greatest increase in M2/M1 polarization (Fig. 6p).

### 2.5 *In vivo* regenerative effects of spheroids encapsulating GC for IR-damaged mouse salivary glands

To ensure the clinical feasibility of our antioxidant carrier, the *in vivo* biocompatibility of 5% and 7% (w/v) GCs was evaluated through comprehensive serum biochemical tests and histological analysis of major organs. 5% and 7% (w/v) GCs were injected into the SGs of mouse, and the animals were sacrificed at 1 week (to assess acute inflammatory response) and 6 weeks (to assess chronic inflammatory response) post-injection.

Fig. S11a and S11b present the serum levels of alanine aminotransferase (ALT) and creatinine, which serve as indicators for hepatic and renal function, respectively. Both the 5% and 7% (w/v) GCs-treated groups showed no significant difference (ns) compared to the Sham group at all time points, demonstrating that the presence and degradation of the GC carrier do not induce metabolic organ damage. Furthermore, systemic safety was qualitatively assessed *via* Hematoxylin and eosin (H&E) staining of metabolic and vital organs (liver, kidney, spleen, heart, lung, and salivary gland) (Fig. S11c). Importantly, histological observations of the local injection site (salivary gland) revealed no signs of tissue necrosis, abnormal inflammatory infiltration, or structural deformities throughout the 6-week study period, confirming the absence of a local inflammatory response. Notably, the previous study utilized the Ru/SPS + blue light system for nerve regeneration and reported no significant inflammatory cell infiltration or tissue necrosis *via* H&E staining results up to 12 weeks post-implantation [33]. This prior evidence, combined with our current findings, confirms that the Ru/SPS + blue light system does not induce *in vivo* toxicity even over extended periods exceeding 6 weeks. Collectively, these findings substantiate that the antioxidant GC carrier is highly biocompatible and systemically safe *in vivo*.

To evaluate the therapeutic effects of spheroids encapsulating GC in IR-damaged SG, a head and neck IR mouse model was established (Fig. S12). C57BL/6 mice were randomly divided into seven groups: Group 1 (sham, non-irradiated control), group 2 (IR only), group 3 (IR + 7% Gel), group 4 (IR + spheroids), group 5 (IR + 7% GC), group 6 (IR + spheroids encapsulating 5% GC), and group 7 (IR + spheroids encapsulating 7% GC). Localized IR to the SG region was performed one day before cell injection (day −1) to induce tissue damage. On day 0, the assigned therapeutic formulations were injected into the periglandular area near the submandibular glands. For the Gel and GC-treated groups, photo-crosslinking was conducted immediately after injection using blue light (450 nm, 1 min) to achieve in situ gelation. Mice were euthanized on day 7 and day 42 for histological, immunohistochemical, and molecular analyses.

#### 2.5.1 In vivo retention and biodistribution of DiR-labeled AdMSC spheroids

**Fig. 7:**
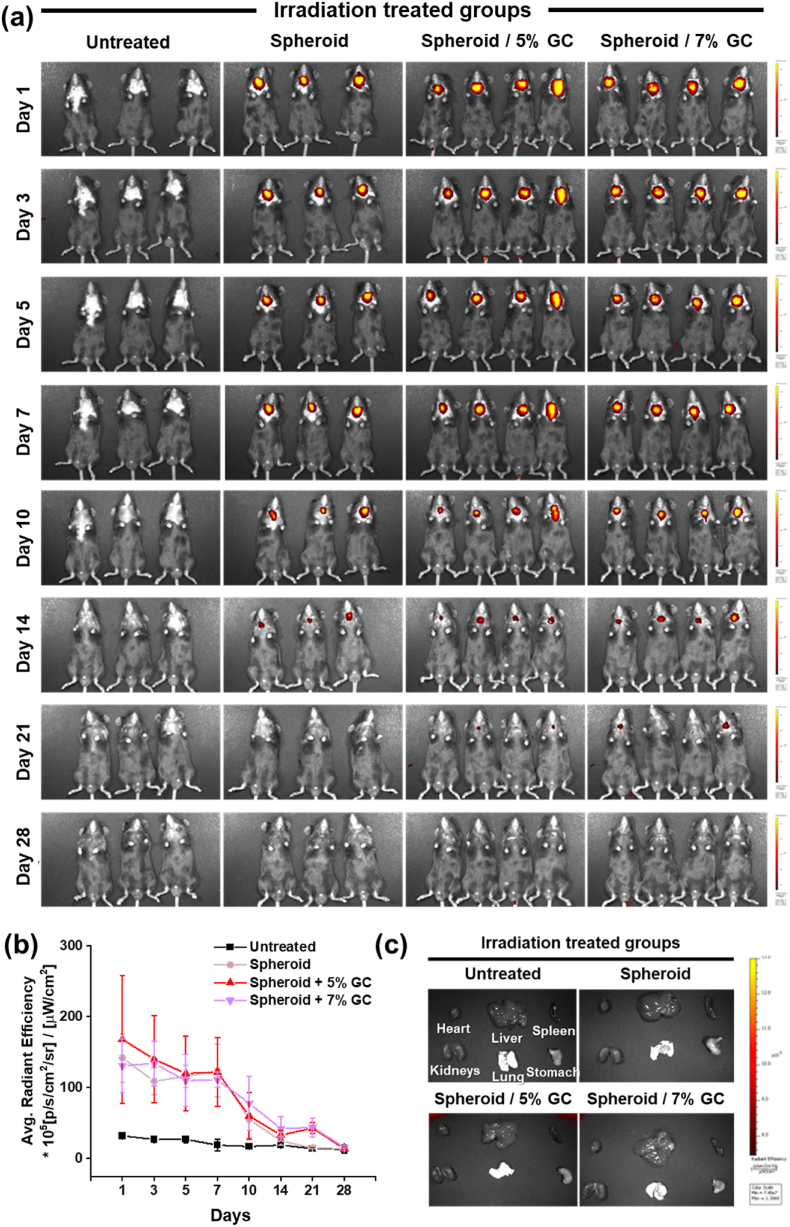
***In vivo* retention and clearance kinetics of DiR-labeled AdMSC spheroids after injection into mouse salivary glands.** (a) DiR-labeled AdMSC spheroids or spheroid + GC (untreated, spheroid, spheroid + GC 5% w/v, spheroid + GC 7% w/v) were injected into the IR-damaged mouse salivary gland, and their fluorescence signals were monitored by IVIS from days 1 to 28 until no detectable signal remained. (b) Quantification of the average radiant efficiency in the salivary gland region over time. (c) At day 28 post-injection, major organs (heart, liver, spleen, kidneys, lungs, and stomach) were harvested and imaged by IVIS to assess DiR signals. Data are presented as mean ± SD (n = 3 or n = 4). Statistical analysis was performed using one-way ANOVA followed by Tukey's post-hoc test. ^ns^*P* > 0.05, ∗*P*≤ 0.05, ∗∗*P*≤ 0.01, and ∗∗∗*P*≤ 0.001.

To evaluate the *in vivo* fate of transplanted AdMSC spheroids, DiR-labeled spheroids and spheroid/GC were injected into the IR-damaged mouse SG, and *in vivo* imaging was performed using IVIS from days 1 to 28. At day 1 post-injection, strong fluorescence signals were detected in the SG region in the spheroid and spheroid + GC groups, whereas only background-level signals were observed in the untreated group (Fig. 7a). In both the spheroid and spheroid/GC groups, the signal intensity gradually decreased over time, and quantitative analysis showed that the average radiant efficiency in the SG region declined most prominently during the first week after injection and approached the detection limit by days 21–28 (Fig. 7b).

At day 28, *in vivo* IVIS imaging no longer detected any apparent DiR signal in whole-body images, and thus mice were euthanized and major organs were harvested for ex vivo imaging. Only background-level, faint signals were detected in other organs such as the heart, liver, spleen, kidneys, lungs, and stomach (Fig. 7c).

#### 2.5.2 Histological evaluation and anti-fibrotic effects of spheroids encapsulating GC

**Fig. 8:**
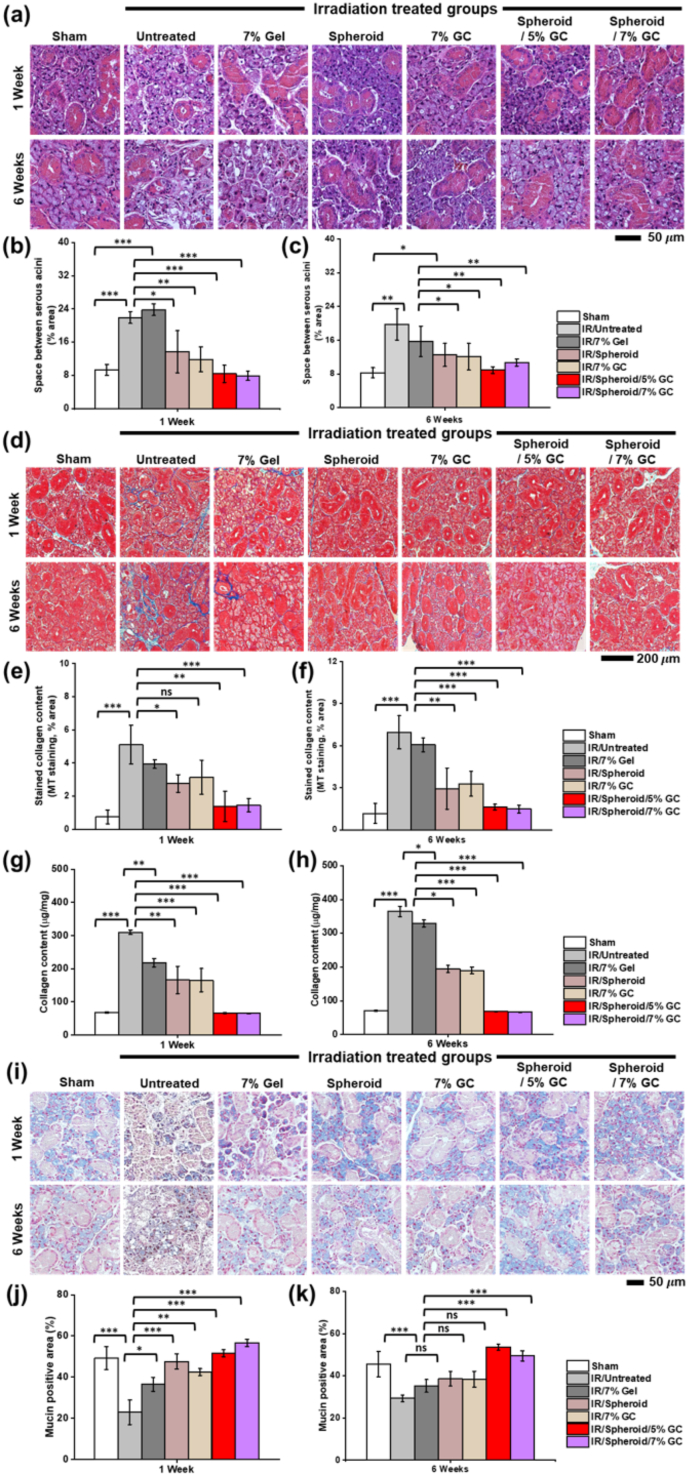
**Histological analysis and anti-fibrotic effects of spheroids encapsulating GC for IR-damaged salivary glands.** (a–c) Hematoxylin and eosin (H&E) staining of SG sections from all groups at 1 and 6 weeks post-IR to evaluate tissue integrity. (d–f) Masson's trichrome (MT) staining to visualize collagen deposition (blue) in tissue sections, indicating fibrosis, at the same time points. (g, h) Quantitative assessment of total collagen content in IR-damaged salivary glands at 1 and 6 weeks post-treatment, measured by a hydroxyproline-based total collagen assay using whole-gland lysates. (i) Alcian Blue staining to detect acidic mucin (blue), reflecting mucin-producing acinar structures were preserved. (j, k) Quantification of mucin-positive area (%) at 1 and 6 weeks. Data are presented as mean ± SD (n = 3). Statistical analysis was performed using one-way ANOVA followed by Tukey's post-hoc test. ^ns^*P* > 0.05, ∗*P*≤ 0.05, ∗∗*P*≤ 0.01, and ∗∗∗*P*≤ 0.001. Scale bars: (a) 50 μm; (d) 200 μm; (i) 50 μm.

Histological analysis using H&E staining revealed significant disruption of acinar cells and ductal structures in groups 2 and 3, indicating extensive tissue damage at 1 week post-IR. In contrast, these structures were notably well preserved in the threated groups, particularly in groups 6 and 7 (Fig. 8a). Quantitative analysis of the space between serous acini (Fig. 8b) showed that groups 2 and 3 had significantly more enlarged inter-acinar spaces than group 1, indicating tissue disruption. Among the treated groups, groups 6 and 7 exhibited the lowest inter-acinar space percentages, suggesting better tissue preservation. As shown in Fig. 8c, at 6 weeks post-treatment, all groups showed a trend toward reduced acinar space. However, groups 6 and 7 maintained significantly smaller inter-acinar spaces than groups 2, 3, 4, and 5, indicating a more efficient and sustained regenerative effect when both spheroids and GC were applied together. MT staining revealed substantially more severe fibrosis in groups 2 and 3, as shown by the increased number of blue-stained collagen fibers in SG tissues (Fig. 8d). In contrast, groups 6 and 7 displayed significantly reduced fibrosis, approaching levels similar to those observed in group 1. This suggests a protective antifibrotic effect in SG tissues treated with the spheroid-encapsulating GC system. This result was further supported by quantitative analyses (Fig. 8e–f). At 1 week, groups 2 and 3 showed the highest levels of collagen deposition, while groups 6 and 7 had significantly lower levels. This trend persisted at 6 weeks, confirming the sustained antifibrotic effects of spheroid-encapsulating GC. Biochemical quantification of total collagen content revealed a marked reduction in collagen levels in the SGs (Fig. 8g–h), and these hydroxyproline-based measurements were consistent with the MT staining scores. Alcian Blue staining revealed marked differences in mucopolysaccharide composition across the experimental groups (Fig. 8i-k). Groups 2 and 3 exhibited a notable reduction in mucin-secreting structures, as indicated by diminished blue staining area, suggesting the loss of both acidic and neutral mucopolysaccharides in IR-damaged SG tissues. In contrast, groups 6 and 7 exhibited strong and uniform purple staining in the acinar regions, resembling the mucin pattern in group 1. This implies preservation of secretory function and mucin composition. These histological findings indicate that the spheroid encapsulating GC system attenuated IR-induced mucosal depletion and supported SG function by restoring mucin-producing components. In addition to histological improvements, functional recovery of the SGs was evaluated. Mice treated with the spheroid-encapsulating GC system (groups 6 and 7) exhibited the greatest recovery in body weight, SG mass, saliva secretion, and total salivary protein concentration compared to other groups (Fig. S13), demonstrating improved systemic and glandular recovery. These results further underscore the therapeutic potential of the spheroid-encapsulating GC system in mitigating IR-induced glandular dysfunction.

#### 2.5.3 Restoration of acinar structure and functions in IR-damaged mouse salivary glands

**Fig. 9:**
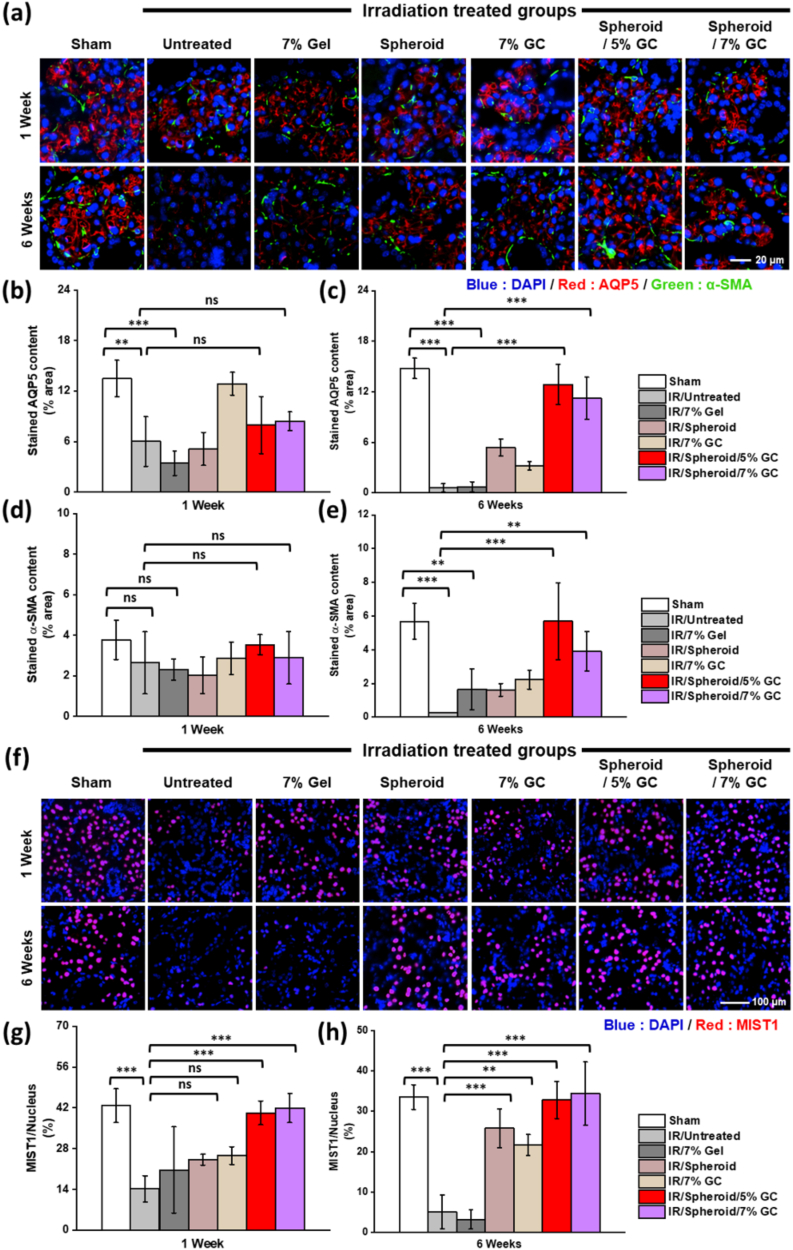
**Restoration of salivary epithelial and acinar markers following spheroid encapsulating GC treatment.** (a–e) Immunofluorescence staining for aquaporin 5 (red, AQP5) and α-smooth muscle actin (green, α-SMA) to label acinar and myoepithelial cells, respectively. (f–h) Immunofluorescence staining for Mist1 (red), a transcription factor associated with acinar cell identity. Data are presented as mean ± SD (n = 3). Statistical analysis was performed using one-way ANOVA followed by Tukey's post-hoc test. ^ns^*P* > 0.05, ∗*P*≤ 0.05, ∗∗*P*≤ 0.01, and ∗∗∗*P*≤ 0.001. Scale bars: (a) 20 μm; (f) 100 μm.

To further validate the preservation of SG structure and function at the protein level, immunofluorescence staining was performed to assess the expression of acinar and myoepithelial markers. A significant reduction in AQP5 (red) and α-SMA (green) expression was observed in groups 2 and 3, indicating that IR caused marked damage to both acinar and myoepithelial components (Fig. 9a). In contrast, groups 6 and 7 displayed well-preserved staining patterns for both markers. Quantitative analysis showed that AQP5 and α-SMA expression levels were significantly higher in groups 6 and 7 than in the irradiated control groups at both 1 week (Fig. 9b–d) and 6 weeks (Fig. 9c–e), indicating sustained preservation or recovery of secretory and structural cell types.

Immunofluorescence staining for Mist1 (red), a marker of secretory acinar cell differentiation, showed markedly reduced nuclear expression in groups 2 and 3 (Fig. 9f). In contrast, groups 6 and 7 exhibited a clear increase in the number of Mist1-positive nuclei. Quantitative analysis confirmed that Mist1 expression levels were significantly elevated in these groups at both 1 week (Fig. 9g) and 6 weeks (Fig. 9h), suggesting effective restoration and long-term maintenance of acinar cell identity following treatment. Interestingly, AQP5 expression in group 5 reached near-sham levels at week 1, indicating rapid functional recovery. However, a marked decrease at 6 weeks suggests that the effect of GC alone may be transient, likely due to the limited duration of its antioxidant activity without sustained paracrine support. Persistent IR-induced damage may have contributed to this decline. Notably, Mist1 expression remained stable in group 5, suggesting that although acinar cell identity was preserved, secretory function (as indicated by AQP5 levels) may not have been maintained.

#### 2.5.4 ROS scavenging and DNA repair capabilities of spheroids encapsulating GC

**Fig. 10:**
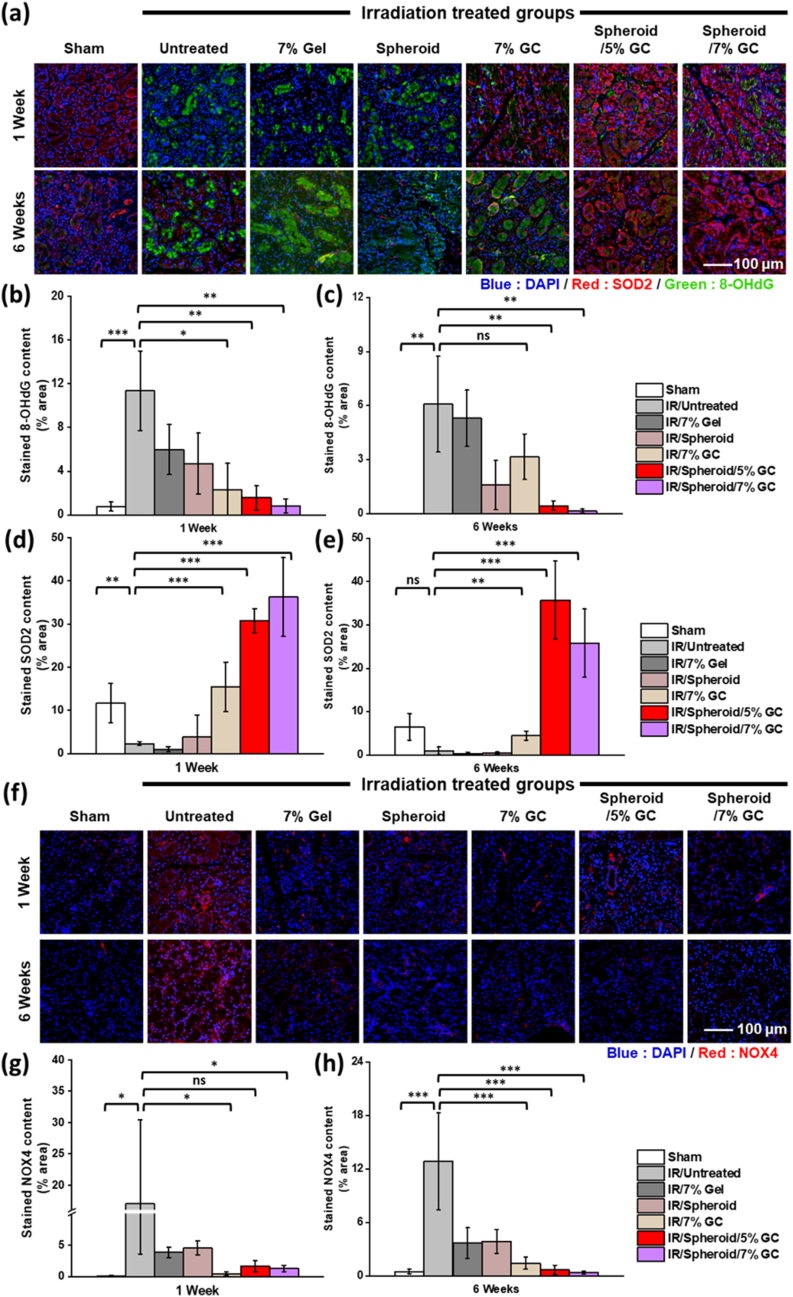
**ROS scavenging and DNA repair capabilities following spheroids encapsulating GC treatment.** (a) Immunofluorescence staining for superoxide dismutase 2 (SOD2; red) and 8-hydroxy-2′-deoxyguanosine (8-OHdG; green) in SG tissue sections to assess antioxidant activity and oxidative damage, respectively. (b, c) Quantification of 8-OHdG-positive areas at 1 and 6 weeks post-IR. (d, e) Quantification of SOD2 expression levels in each group at both time points. (f) Immunofluorescence staining for NADPH oxidase 4 (NOX4; red), a marker of ROS-induced damage, in SG sections. (g, h) Quantitative analysis of NOX4-positive areas at 1 and 6 weeks. Data are presented as mean ± SD (n = 3). Statistical analysis was performed using one-way ANOVA followed by Tukey's post-hoc test. ^ns^*P* > 0.05, ∗*P*≤ 0.05, ∗∗*P*≤ 0.01, and ∗∗∗*P*≤ 0.001. Scale bars: (a) 100 μm; (f) 100 μm.

Immunohistochemical analysis was conducted to evaluate oxidative stress and ROS-induced DNA damage using SOD2 as an antioxidant marker and 8-OHdG as a marker of oxidative DNA damage marker (Fig. 10a). After 1 week, groups 5, 6, and 7 exhibited elevated SOD2 expression, indicating that both GC and spheroids contributed to enhanced ROS scavenging. By week 6, groups 6 and 7 demonstrated lower 8-OHdG expression than that on week 1, while SOD2 levels remained elevated. In contrast, group 5 showed reduced SOD2 expression at 6 weeks and group 4 had persistently lower 8-OHdG expression, suggesting that long-term DNA repair was primarily driven by the presence of spheroids (Fig. 10b–e). This finding was further supported by NOX4 analysis, another marker of ROS-induced DNA damage (Fig. 10f). At 6 weeks, NOX4 expression was lower than that at 1 week in group 4. Notably, groups 6 and 7 exhibited significant reductions in NOX4 expression at 6 weeks relative to week 1 (Fig. 10g–h). These results collectively suggest that the spheroids encapsulated in GC effectively support both ROS scavenging and long-term DNA repair in IR-damaged SG.

#### 2.5.5 Angiogenic effect of spheroids encapsulating GC in IR-damaged mouse salivary glands

**Fig. 11:**
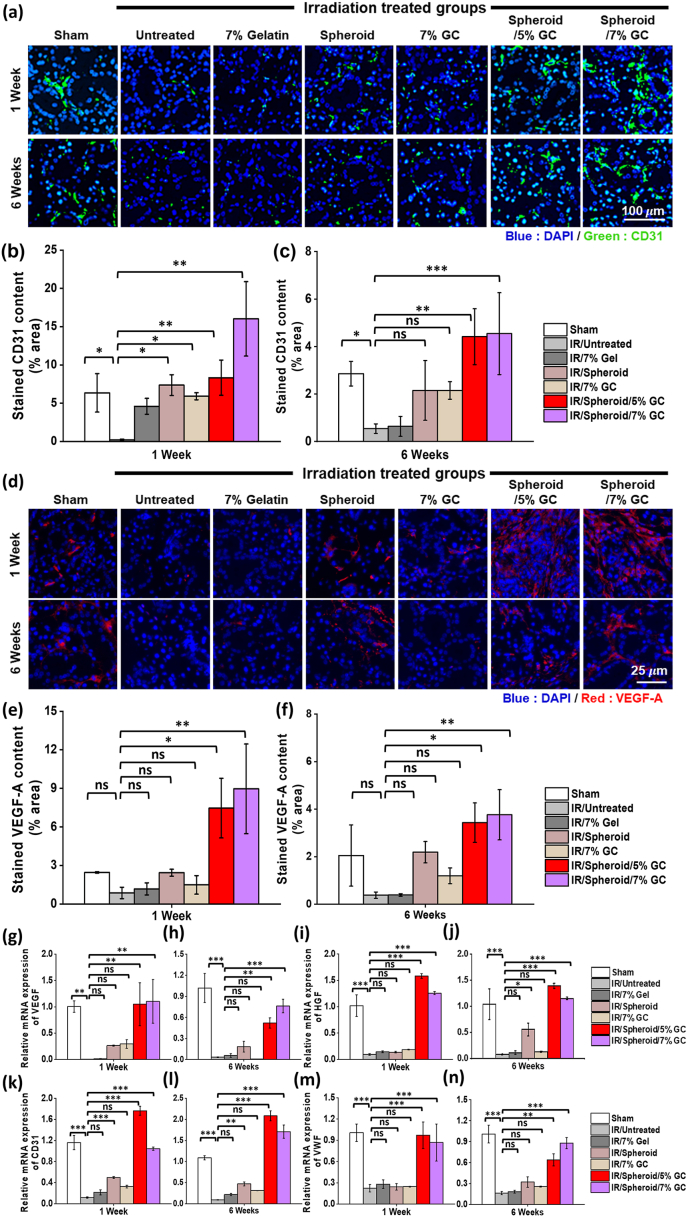
**Angiogenic effect of spheroids encapsulating GC for IR-damaged salivary glands.** (a) Immunofluorescence staining for CD31 (green), an endothelial cell marker, in SG tissue sections to evaluate neovascularization. (b, c) Quantification of CD31-positive areas at 1 and 6 weeks post-treatment. (d) Immunofluorescence staining for VEGF-A (red), an angiogenesis marker, in SG tissue sections. (e, f) Quantification of VEGF-A-positive areas at 1 and 6 weeks post-treatment. (g–n) Relative mRNA expression levels of angiogenesis-related genes, including VEGF, HGF, CD31, and vWF, in mouse SG tissues harvested at 1 and 6 weeks post-treatment, as determined by quantitative PCR. Data are presented as mean ± SD (n = 3). Statistical analysis was performed using one-way ANOVA followed by Tukey's post-hoc test. ^ns^*P* > 0.05, ∗*P*≤ 0.05, ∗∗*P*≤ 0.01, and ∗∗∗*P*≤ 0.001.

To evaluate vascularization induced by the spheroids, CD31 expression, an endothelial cell marker, was assessed at 1 and 6 weeks post-treatment (Fig. 11a). CD31-positive areas (green) indicate endothelial cells involved in neovascularization, with nuclei counterstained with DAPI (blue). At 1 week, CD31 expression was significantly lower in group 2 than in group 1, confirming IR-induced vascular damage. In contrast, groups 6 and 7 exhibited markedly higher CD31 expression among the IR-damaged groups. By 6 weeks, CD31 levels were further elevated in groups 6 and 7, suggesting sustained angiogenic activity. Quantitative analysis confirmed significant increases in CD31-positive areas in group 6 and group 7 at 1 week and 6 weeks (Fig. 11b–c), indicating that spheroid delivery *via* GC promoted both early and long-term vascular regeneration in irradiated SG tissue. Consistently, H&E-based assessment of microvessel density also showed higher numbers of small vessels in the spheroid + GC groups compared with irradiated controls (Fig. S14).

VEGF-A immunofluorescence staining was performed to evaluate changes in angiogenic factor expression in IR-damaged SGs. Group 6 and 7 exhibited markedly increased VEGF-A-positive signals at both 1 and 6 weeks compared with the IR only and IR + 7% gelatin groups. These findings indicate that the spheroid + GC systems effectively enhance local VEGF-A protein expression in IR-damaged SGs (Fig. 11d–f).

To further corroborate these histological findings at the molecular level, quantitative PCR was performed using RNA extracted from irradiated mouse SG tissues harvested at 1 and 6 weeks post-treatment. Consistent with the CD31 immunostaining data, the expression of angiogenesis-related genes, including VEGF, HGF, CD31, and vWF, tended to decrease after IR but was restored or further upregulated in the spheroid + GC groups, particularly in groups 6 and 7 (Fig. 11g–n). These results suggest that spheroid encapsulating GC enhances neovascularization in IR-damaged SG not only by increasing endothelial cell content but also by upregulating key pro-angiogenic factors at the transcriptional level.

#### 2.5.6 Anti-apoptotic efficacy of spheroids encapsulating GC in IR-damaged mouse salivary glands

**Fig. 12:**
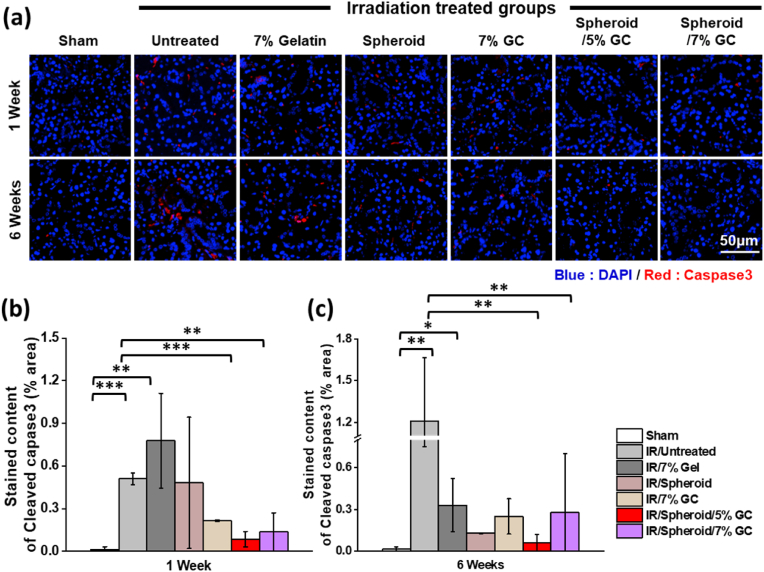
**Anti-apoptotic efficacy of spheroids encapsulating GC following IR.** (a) Immunofluorescence staining of cleaved caspase-3 (red), a marker of apoptosis, in salivary gland sections from different treatment groups. Nuclei were counterstained with DAPI (blue). (b, c) Quantification of cleaved caspase-3–positive areas at 1 and 6 weeks post-IR. Data are presented as mean ± SD (n = 3). Statistical analysis was performed using one-way ANOVA followed by Tukey's post-hoc test. ^ns^*P* > 0.05, ∗*P*≤ 0.05, ∗∗*P*≤ 0.01, and ∗∗∗*P*≤ 0.001.

Cleaved caspase-3 is a well-established marker of apoptosis, representing the activated form of caspase-3 involved in the execution phase of programmed cell death. Immunofluorescence staining for cleaved caspase-3 revealed prominent apoptosis in group 2 at both 1 and 6 weeks post-IR, with a substantial number of cleaved caspase-3–positive cells observed (Fig. 12a). In contrast, groups 5, 6, and 7 exhibited markedly reduced cleaved caspase-3 expression, with group 7 displaying a pattern comparable to that of group 1. Quantitative analysis confirmed a significant reduction in the cleaved caspase-3–positive area in group 7, indicating the strong anti-apoptotic effect of the treatment (Fig. 12b–c).

## 3 Discussion

This study presents a novel strategy to restore irradiated SGs by leveraging the synergistic benefits of AdMSC spheroids and an antioxidant GG-based carrier. While AdMSC spheroids have shown promise in regenerative medicine, their delivery and survival in the harsh post-irradiation environment remain major challenges. Here, we addressed these limitations by developing a thiol-rich gelatin hydrogel that provides spheroid protection and enhances regenerative outcomes through both antioxidant action and tissue-supportive functions.

Conventional approaches to mitigating IR-induced damage primarily rely on short-lived antioxidant molecules, which are insufficient to counter prolonged oxidative stress. Although strategies, such as dopamine-conjugated hydrogels, have attempted to improve retention, they often lack the biological functionality and long-term stability needed for effective tissue regeneration [34,35]. In this study, we synthesized thiol-rich gelatin through EDC/NHS coupling, enabling sustained antioxidant activity. This chemical functionalization overcame the short half-life of GSH and ensured persistent ROS-scavenging capacity in IR-damaged environments. Unlike natural carriers, such as Matrigel or HA hydrogels, which suffer from limited structural integrity, rapid degradation, and insufficient biological functionality *in vivo*, GC offers stable spheroid encapsulation and prolonged antioxidant support under oxidative conditions. Notably, GC maintained its antioxidant capacity even after photo-crosslinking, as demonstrated by concentration-dependent increases in total antioxidant capacity and sustained DPPH radical scavenging activity. Despite potential oxidation risks under blue light, thiol groups within GG remained functionally preserved. Additionally, intracellular ROS suppression in H_2_O_2_-treated cells demonstrated that GC can effectively neutralize ROS in a prolonged oxidative stress environment, outperforming free GSH.

In addition to its antioxidant properties, GC exhibited tunable mechanical characteristics depending on GG concentration, enabling stable cell encapsulation and localized delivery. Its biocompatibility and functional durability make GC a promising platform for cell-based therapies targeting IR-induced damage and other ROS-related diseases, although long-term *in vivo* safety and clearance of the photocatalysts (Ru and SPS) still require further investigation. Among the tested concentrations, 5% (w/v) GC produced the most favorable biological responses, with enhanced cell proliferation within the GC and extensive outward spreading, whereas 7% (w/v) GC yielded the highest VEGF secretion, indicating that a balance between mechanical softness and structural integrity is critical for therapeutic efficacy.

AdMSC spheroids fabricated in agarose molds were well formed and size-uniform, and they secreted substantially higher levels of VEGF and HGF than 2D-cultured cells (same number of monolayer cultured AdMSCs), confirming a clear paracrine advantage of the 3D configuration. In the context of radiation-induced salivary gland injury, VEGF is expected to drive revascularization of damaged tissues, whereas HGF has been implicated in promoting salivary gland branching morphogenesis and protecting against radiation-induced dysfunction by activating pro-survival and anti-apoptotic pathways in salivary epithelial and progenitor cells. Thus, the increased VEGF and HGF secretion from AdMSC spheroids in our system supports the notion that spheroid formation augments not only angiogenesis but also epithelial protection and regeneration in irradiated salivary glands [36,37]. Upon encapsulation in GCs, spheroids experienced a mechanically permissive yet structurally supportive niche that allowed cells to spread outward while maintaining and further amplifying their paracrine activity. Also, a sufficient network structure to provide mechanical integrity even as the GC underwent degradation over 5 days (Fig. S9g).

Moreover, analysis of Fig. S9d–S9f further supports that VEGF upregulation is not determined by spheroid size alone. In our system, hypoxic core staining demonstrates that compact spheroids initially exhibit a marked hypoxic region, which gradually diminishes as cells spread outward within the gelatin-based hydrogels and the spheroidal architecture becomes partially lost, particularly in the 5-7% (w/v) GCs. Nevertheless, conditions exhibiting greater spheroid spreading and reduced apparent diameter (particularly 5-7% w/v GCs) showed higher cumulative VEGF secretion, and VEGF levels also increased with GC concentration over time, despite the attenuation of hypoxic staining and partial loss of compact 3D architecture. Moreover, even in the TCPS group, where initially formed spheroids progressively transition toward a more 2D-like configuration, VEGF expression remains elevated while many cells spread across the dish surface. These findings suggest that initial hypoxic priming during 3D spheroid formation induces a durable pro-angiogenic phenotype that persists as cells redistribute and engage more extensively with the surrounding matrix or substrate, and that GC-mediated modulation of the microenvironment (antioxidant and mechanical cues) further amplifies VEGF production beyond what can be explained by spheroid size or hypoxic core extent alone.

Functionally, spheroid + GC system more strongly promoted endothelial tube formation and protected cells from H_2_O_2_-induced apoptosis than spheroids or soluble VEGF alone, indicating that GC stabilizes spheroids and converts their secretome into enhanced angiogenic and cytoprotective outcomes. In addition to their angiogenic and cytoprotective effects, spheroid encapsulating GCs also modulated the inflammatory microenvironment in a manner consistent with a pro-regenerative MSC phenotype (Fig. S15). Consistent with these *in vitro* findings, histological and functional analyses demonstrated antioxidant activity, structural support, angiogenesis, and apoptosis inhibition *in vivo*, together with preservation of acinar and ductal architecture, reduced fibrosis, and recovery of mucin secretion. Overall, well-formed AdMSC spheroids within mechanically optimized GCs how substantial potential to improve engraftment and functional recovery, and these findings should be considered alongside the subsequent discussion of immunological features, limitations of the *in vivo* model, and the translational implications for clinical application.

The spheroid + GC system also exhibited advantages *in vivo*. DiR-based IVIS imaging showed that DiR-labeled spheroid + GC groups maintained fluorescence at the injection site for a longer period than spheroids alone, indicating that the GC matrix prolonged local spheroid retention. This extended retention likely provided a temporal window for sustained paracrine activity, which could contribute to the long-term structural and functional recovery of the IR-damaged salivary gland. Moreover, the gradual decline in fluorescence toward the detection limit by day 28 indicates that the grafts are not permanently retained but are progressively cleared, and the fact that no DiR signal was detected in any other organs at the end of the observation period suggests a low risk of long-term adverse effects related to residual implanted material.

Sustained SOD2 expression and decreased levels of ROS-induced damage markers, such as 8-OHdG and NOX4, indicate that the spheroids + GC system not only mitigates early oxidative stress but also promotes long-term DNA repair [[38], [39], [40]]. These molecular effects are likely key contributors to functional recovery and the prevention of chronic tissue degeneration. Furthermore, reduced cleaved caspase-3 expression in GC-treated groups points to enhanced apoptosis resistance, supporting prolonged tissue preservation [41]. Complementing these effects, the observed shift toward M2 macrophage polarization demonstrates that the spheroids + GC system also modulates the immune microenvironment in a pro-regenerative direction (Fig. S15).

Despite these promising findings, certain limitations should be addressed. This study was conducted in a mouse model and focused on elucidating the mechanisms and therapeutic effects of the spheroid-encapsulating GC system in IR-damaged SGs. Future work will be required to validate these benefits in larger animal models and, ultimately, in humans, in order to optimize dosing, delivery strategies, and long-term safety.

Furthermore, the current delivery method involved surgical exposure of the SG, which may hinder direct clinical translation. For human applications, ultrasound-guided intraglandular injection would be essential to enable minimally invasive delivery. Future study will aim to evaluate spheroids encapsulating GC in larger animal models to determine the optimal balance between mechanical support and biological activity. Additionally, this therapeutic system showed strong potential for broader application across various ROS-related pathologies, including ischemic injuries, chronic wounds, and inflammatory diseases, where oxidative stress plays a central role. The combined benefits of the sustained antioxidant activity of GC and the paracrine effects of AdMSC spheroids may lay the foundation for adaptable and customizable therapies tailored to diverse tissues and clinical scenarios. The structural stability and functional durability of GC further support its translational potential for human therapy.

In summary, this study presents a transdisciplinary approach that integrates a biomaterial platform capable of ROS scavenging, oxidative stress suppression, and cell delivery, with AdMSC spheroids that contribute to immune modulation and tissue repair. By leveraging the physical and biological functionalities of GC and the regenerative capacity of AdMSC spheroids, we demonstrate strong translational potential for IR-damaged SG regeneration. Importantly, although previous studies have focused on either paracrine support or structural protection alone, our combined strategy provides synergistic advantages in microenvironmental conditioning and cellular function, leading to robust and sustained regenerative outcomes.

Additional statistical analyses restricted to the treatment groups in the *in vivo* studies revealed consistent trends favoring the spheroid + GC combination over monotherapy. For the main functional and histological endpoints presented in this work, these differences reached conventional statistical significance and are reported as such. In contrast, some supplementary comparisons did not achieve significance, likely owing to the limited sample size and inter-individual variability; these latter data were therefore regarded as supportive trends rather than definitive evidence of head-to-head superiority and were not all included in the main figures.

## 4 Conclusion

A multifunctional therapeutic system was developed by AdMSC spheroid encapsulating thiol-rich hybrid protein-based cell carrier to treat IR-induced salivary gland dysfunction. This antioxidant GC platform provided injectability, mechanical support, and sustained ROS scavenging while maintaining a hypoxia-primed 3D spheroid niche with enhanced paracrine secretion of angiogenic, cytoprotective, and immunomodulatory factors. In an IR-damaged mouse model, spheroid encapsulating GC improved local retention and promoted salivary gland structural and functional recovery by reducing oxidative stress, fibrosis, and apoptosis and augmenting VEGF-driven angiogenesis. Together, these results identify AdMSC spheroids within antioxidant GC as a translatable strategy for xerostomia and other oxidative stress–mediated tissue disorders.

## 5 Experimental section

### *5.1* Synthesis of glutathione-conjugated gelatin (GG) hybrid protein

A biocompatible and simple 1-ethyl-3-(3-dimethylaminopropyl) carbodiimide/N-hydroxysuccinimide (EDC/NHS) coupling method was used to synthesize glutathione (GSH, Sigma-Aldrich)-conjugated gelatin (Type A from porcine skin, Sigma-Aldrich)-conjugated hybrid proteins. Gelatin was dissolved in 0.5 M MES buffer (pH 6, BYLABS) to prepare a carboxyl-activated gelatin solution. The solution was stirred magnetically at 36 °C until completely dissolved. Next, a 100 mM EDC (Sigma-Aldrich) solution was added and reacted at 36 °C for 10 min, followed by the addition of 200 mM NHS (Sigma-Aldrich). Then, a 40 mM GSH solution was added dropwise. Amide bond formation between the carboxyl groups of Gel and the primary amine groups of GSH was induced under an argon atmosphere at 36 °C with continuous magnetic stirring for 2 h. To remove unreacted reagents, dialysis was performed in deionized (DI) water for 3 d, with the medium changed three times daily. After 1 week of lyophilization, the GG hybrid protein was obtained in powder form.

### 5.2 Confirmation of GG hybrid protein synthesis

FT-IR spectroscopy (NICOLET iS10 spectrometer; Thermo Fisher Scientific, USA) was used to confirm successful amide bond formation in the GG. Characteristic peaks for gelatin, GSH, and GG were recorded in attenuated total reflectance mode. Measurements were taken over a range of 500–4000 cm^−1^ by performing 16 scans per sample.

To evaluate changes in secondary protein structure, CD spectra were acquired using a circular dichroism spectropolarimeter (J-815; Jasco, Japan). Gel and GG were each prepared at a concentration of 0.1 mg mL^−1^ in 1X phosphate-buffered saline (PBS, pH 7.4, Thermo Fisher Scientific). A 400 μL volume of each solution was placed in a quartz cell with a 1-mm path length. Spectra were recorded in the far-UV region (190–250 nm) under flowing nitrogen gas.

Thiol group content in the GG backbone was quantified using Ellman's assay. Powdered 5,5′-dithio-bis-(2-nitrobenzoic acid) (DTNB; Thermo Fisher Scientific) and standard GSH were each dissolved in 0.1 M sodium phosphate buffer (pH 8.0, 1 mM EDTA, BYLABS) and 1X PBS, respectively. The GSH standard and DTNB solutions were mixed at a 1:1 vol ratio in a well plate, and absorbance was measured at 412 nm using a microplate reader (VersaMax; Molecular Devices, USA) to generate a standard curve. To assess thiol content in the GG samples, 1%, 3%, 5%, and 10% (w/v) GG solutions and a 1% (w/v) Gel solution were prepared in 1X PBS. Each sample was mixed with an equal amount of DTNB solution and thoroughly mixed in a well plate. Absorbance was measured at 412 nm, and thiol concentrations were calculated based on the GSH standard curve.

### 5.3 Antioxidant capacity of GG hybrid protein

The antioxidant activity of the GG hybrid protein was assessed using the DPPH radical scavenging assay (Dojindo) and the total antioxidant capacity test (Biomax), following the manufacturer's protocols. The radical scavenging ability and overall antioxidant capacity of GG were quantitatively evaluated by measuring absorbance at 517 nm and 460 nm, respectively, using a microplate reader.

### 5.4 Fabrication of GC

To form a stable network for AdMSC spheroid encapsulation, covalent bonding between tyrosine residues in the GG matrix was induced. A homogeneous solution of 3–7% (w/v) GG was prepared in 1X PBS with magnetic stirring at 37 °C. Photocatalyst Tris (2,2′-bipyridyl)dichloro-ruthenium-2-hexahydrate (Ru, Sigma-Aldrich) and sodium persulfate (SPS, Sigma-Aldrich) were each dissolved in 1X PBS to obtain 1 mM Ru and 30 mM SPS solutions, respectively. These solutions were added dropwise to the GG solution under continuous stirring. The resulting GG-photocatalyst mixture was chemically crosslinked *via* blue light exposure (450 nm, dental curing light) for 1 min, forming GG-based carrier.

### 5.5 Physical properties of GC

The GC was fabricated using a biocompatible photo-crosslinking system, resulting in the formation of di-tyrosine bonds between tyrosine residues in the GG network. The intrinsic autofluorescence of these di-tyrosine groups was utilized as a quantitative indicator to measure the crosslinking density of the hydrogel. For quantification, 100 μL of each GC precursor solution (3-7% w/v) was loaded into each well of a black 96-well plate, and crosslinked under blue light conditions. The fluorescence intensity was measured using a microplate reader (excitation/emission: 280/325 nm). The raw fluorescence intensity values obtained directly from the instrument were utilized to construct the hydrogel network density graphs without any additional normalization, ensuring that the plotted data accurately represent the relative crosslinking density across the different GC groups.

To determine Gel content, 3–7% (w/v) GC samples (100 μL) were prepared and freeze-dried to obtain the initial weight (W_i_). The samples were then immersed in 3 mL of 1X PBS and incubated at 37 °C for 24 h to remove unreacted components. After drying and freeze-drying again, the final weight (W_f_) was measured. Gel content (%) was calculated using Equation (1):

(1) $$\text{Gel}\;\text{content}\;\left(\%\right)=\left(W_{f}/W_{i}\right)\times 100\%$$

To evaluate the swelling behavior, GC samples [100 μL of 3–7% (w/v)] were weighed to determine the initial weight (W_i_). Then, they were immersed in 1 mL of 1X PBS. At predetermined time points (1–7 d), the swollen weight (W_t.p_) was recorded. The swelling ratio (%) was calculated using Equation (2):

(2) $$\text{Swelling}\;\text{ratio}\;\left(\%\right)=\left(\left(W_{t.p}-W_{i}\right)/W_{i}\right)\times 100$$

### 5.6 Mechanical and rheological properties of GC

The mechanical strength of GC was analyzed using a universal testing machine (Instron 5966; Instron Corp., USA) equipped with a 10 N load cell. Compression testing was performed at a crosshead speed of 5 mm/min.

The viscoelastic properties of GC were analyzed using a rheometer (MCR102e; Anton Paar, Austria) with a 20 mm diameter plate and a 0.5 mm gap. Frequency sweep tests were performed across a range of 1–10 Hz at a constant strain of 1% at 25 °C. To evaluate the shear-thinning behavior, the dynamic viscosity of the GCs was measured as a function of the shear rate, ranging from 0.1 to 1000 s^−1^ at 25 °C.

### 5.7 Biocompability of GC

#### 5.7.1 Qualitative Cck-8 assay

To assess biocompatibility, L929 cells (P573; Korean Cell Bank, Seoul, Republic of Korea), a standard model for cytotoxicity evaluation of biomaterials, were used. GC solutions (3–7% (w/v), 70 μL) were prepared and incubated in 1 mL of Roswell Park Memorial Institute (RPMI)-1640 medium (Thermo Fisher Scientific) supplemented with 10% (v/v) fetal bovine serum (FBS, Thermo Fisher Scientific) and 1% (v/v) penicillin-streptomycin (PS, Thermo Fisher Scientific) at 37 °C in 5% CO_2_ for 24 h. On the following day, the resulting GC extracts were sterilized *via* syringe filtration and added (200 μL) to L929 cells seeded at 1 × 10^3^ cells/200 μL. Cells were cultured for 3 d. At each time point, 150 μL of cell counting kit-8 reagent (CCK-8, Dojindo) was added to each well and incubated for 2 h at 37 °C under 5% CO_2_. Absorbance was measured at 450 nm using a microplate reader to assess cell viability.

#### 5.7.2 Flow cytometry

To quantitatively evaluate cell membrane integrity and apoptosis marker expression, all samples were harvested, washed, fixed and permeabilized using Cytofix/Cytoperm kit (BD Biosciences). Subsequently, cells were stained with Zombie Aqua (1:1000, BioLegend) and Cleaved Caspase-3 (1:5000, Cell signaling technology). The fluorescence intensity of the stained cells was measured using a flow cytometer (BD LSRFortessa™ Cell Analyzer, BD Biosciences, USA).

#### 5.7.3 Live and dead assay

To evaluate cell morphology and viability, Live/Dead staining (Thermo Fisher Scientific) was performed. On days 3, 100 μL of staining solution (calcein-acetoxymethyl and ethidium homodimer-1 mixture) was added to each well and incubated at 37 °C under 5% CO_2_ for 15 min. Stained cells were visualized using a confocal laser scanning microscope (Leica TCS STED CW; Leica Camera AG, Germany).

#### 5.7.4 Immunostaining

For immunostaining, all samples were fixed with 4% paraformaldehyde (Biosesang) at 25 °C for 10 min. Subsequently, permeabilization was performed with 0.3% TritonX-100 (Biosesang) at 25 °C for 5 min. To block non-specific binding, all samples were incubated with 4% bovine serum albumin (BSA) solution for 1 h at 25 °C. Cells were then treated with primary anti-caspase-3 antibody (1:100, Santa Cruz Biotechnology) and anti-cleaved caspase-3 antibody (1:400, Cell signaling technology) for 2 h in a dark environment at 25 °C. Subsequently, the secondary antibody (Alexa Fluor®488 for Cleaved Caspase-3, 1:200 & Alexa Fluor®594 for Caspase-3, 1:1000) was treated for 1 h in a dark environment at 25 °C. Cell nuclei were stained using mounting medium with DAPI (Abcam). All samples were analyzed using a confocal laser scanning microscope.

### 5.8 Antioxidant properties of GC

#### 5.8.1 DPPH radical scavenging test

The antioxidant function of GC was initially evaluated using a DPPH radical scavenging assay, following the manufacturer's instructions.

#### 5.8.2 The sustained antioxidant capacity test

To evaluate the sustained antioxidant efficacy and half-life overcome of GC, dialysis membrane bags containing 10 mM GSH solution and 5-7% (w/v) GC were immersed in 1 mL of 500 μM H_2_O_2_ solution at 37 °C, with the H_2_O_2_ solution refreshed daily to maintain chronic oxidative stress. Extracts were harvested at pre-determined intervals (Days 1, 3, and 7) and subsequently applied to L929 (5.0 × 10^3^ cells) and salivary gland epithelial cells (5.0 × 10^3^ cells) previously seeded in 96-well plates. After a 24 h incubation period in a incubator (37 °C, 5% CO_2_), the remining antioxidants were reacted with CCK-8 reagents, where the optical density was measured at 450 nm using microplate reader.

#### 5.8.3 Intracellular ROS level evaluation

To assess intracellular ROS protection, NIH3T3 mouse embryonic fibroblasts (P184, 2.0 × 10^4^ cells/200 μL; Korean Cell Bank) were treated with various concentrations of hydrogen peroxide (H_2_O_2_; 50, 250, 500, 1000 μM, Thermo Fisher Scientific) to induce oxidative stress. Cell viability was measured to determine the optimal concentration. A 500 μM H_2_O_2_ solution, prepared by diluting 9.8 M H_2_O_2_ in RPMI medium, was selected for the intracellular ROS assay. GC samples [5–7% (w/v)] were incubated in 500 μM H_2_O_2_-containing RPMI medium at 37 °C for 24 h. The H_2_O_2_-treated GC extracts were sterilized *via* syringe filtration and applied to NIH3T3 cells and salivary gland epithelial cells. The intracellular ROS levels were measured using the 2′,7′-dichlrorofluorescence cellular ROS detection assay kit (DCFDA, Abcam), according to the manufacturer's instructions. Fluorescence intensity from the H_2_O_2_-treated NIH3T3 cells or salivary gland epithelial cells (positive control) and GC-treated cells were recorded as a percentage relative to the NC (untreated NIH3T3 cells or salivary gland epithelial cells) to evaluate the protective antioxidant effects of the GC.

#### 5.8.4 H_2_O_2_ scavenging activity

To quantify the concentration-dependent antioxidant performance of GC, an H_2_O_2_ scavenging assay was performed using 5-7% (w/v) GCs. Each GC was immersed in H_2_O_2_ solutions of varying concentrations (500 μM, 2 mM, and 5 mM) and incubated at 37 °C for 24 h to allow for a comprehensive reaction between the chemically conjugated thiol groups and the ROS molecules. After incubation, the supernatants were collected, and the residual H_2_O_2_ levels were measured using a commercial H_2_O_2_ assay kit (Abcam) according to the manufacturer's instructions.

#### 5.8.5 Flow cytometry

For experiments, 5-7% (w/v) GC (100 μL) were prepared and then incubated in 500 μM H_2_O_2_ containing complete media for 7 days. After that, the supernatants were collected and applied to pre-seeded salivary gland epithelial cells (5.0 × 10^5^ cells/well) in a 6-well plate, followed by reaction for 1 day. Cells were then harvested, washed with cold PBS, and stained with a primary antibody (Cleaved Caspase-3, Cell signaling technology) and secondary antibody (Cleaved Caspase-3 Alexa Fluor™ 488 donkey, Invitrogen) according to the manufacturer's instructions, respectively. Flow-cytometric analysis was performed using a BD FACSCanto™ Clinical Flow Cytometry System (BD Biosciences, USA) in Flow Cytometry Core Facility of the Center for Medical Innovation (CMI), Seoul National University Hospital, and the apoptotic cells were quantified.

### 5.9 Formation of spheroids

Human AdMSCs were obtained using the StemPro Human Adipose-Derived Stem Cell Kit (Cat. No. R7788110) and cultured in DMEM (Gibco, Cat.No.11995-065) supplemented with 10% FBS (Gibco, Cat. No. 16000044), 20 ng mL^−1^ human FGF recombinant protein (Peprotech NJ, Cat. No. AF-100-18B-1 MG), 20 ng mL^−1^ human EGF recombinant protein (Peprotech NJ, Cat. No. AF-100-15-1MG), and 1% PS (10,000 U mL^−1^) (Gibco, Cat. No. 15140122). Cells were incubated at 37 °C in a 5% CO_2_ atmosphere.

To generate spheroids, Micro Tissues® 3D Petri Dish micro-molds (Cat. No. Z764000) were used. A 1.5% (w/v) agarose solution (UltraPure Agarose; Invitrogen, Cat. No. 16500100) was prepared in PBS (Gibco, Cat. No. 10010023) and sterilized by autoclaving. The sterilized agarose solution was transferred to a fume hood and stirred using a magnetic bar on a hot plate at 80 °C during mold preparation. The molten agarose was then pipetted into the molds and allowed to solidify on an ice pack for 5 min. After solidification, the agarose molds were transferred to individual wells of a 24-well plate, immersed in PBS, and stored at 4 °C until further use. AdMSCs were seeded into each mold at a density of 1 × 10^6^ cells per mold and cultured in AdMSC growth medium.

### 5.10 AdMSC spheroid diameter measurement

Throughout the 3-day culture period, AdMSC spheroids formed on agarose molds placed in a 12-well plate were imaged daily using a bright-field microscope. The Feret diameter of individual spheroids was measured using ImageJ software (NIH, USA), enabling quantitative evaluation of spheroid size.

### 5.11 Hypoxic core staining of AdMSC spheroids

To evaluate hypoxic core formation, AdMSC spheroids cultured for 3 days were transferred to glass-bottom dishes and stained using an Image-iT Green Hypoxia Reagent (Invitrogen, I14834) according to the manufacturer's instructions. For spheroid culture, the working solution was added directly to the existing culture medium to achieve a final concentration of 1 μM, and samples were incubated for 60 min at 37 °C in a incubator to allow probe diffusion and hypoxia-dependent activation. After incubation, spheroids were gently rinsed 2 times with PBS to reduce background fluorescence, nuclei were counterstained with DAPI (Thermo Scientific™, Cat. No. D1306), and immediately imaged using a EVOS M700 microscope (Invitrogen) with a 488 nm excitation laser and a FITC/GFP emission filter set.

### 5.12 Comparison of paracrine factor secretion in 2D and spheroid cultures

For 2D culture, AdMSCs were seeded in 12-well plates at 5 × 10^5^ cells per well and cultured for 24 h before medium collection. For spheroid culture, pre-formed AdMSC spheroids (approximately 300 μm in diameter) were maintained in agarose molds under identical medium conditions. Conditioned media from both 2D and spheroid cultures were collected stored at −80 °C. VEGF and HGF levels were quantified using human VEGF (ABclonal, RK00023) and human HGF ELISA kits according to the manufacturers’ protocols, and absorbance was read at 450 nm with 540 nm reference using a microplate reader (VersaMax; Molecular Devices, USA). Cytokine concentrations were calculated from standard curves and normalized to cell number or spheroid number where indicated.

### 5.13 Morphological changes of spheroids in GC

After 3 days of culture in agarose molds, during which spheroids formed, GG precursor solutions at concentrations of 4–7% (w/v) were added 100 μL directly to the molds containing spheroids. Crosslinking was initiated by exposure to 450 nm blue light for 1 min. Following encapsulation, spheroids were cultured for an additional 5 days in AdMSC growth medium. The systems were then washed twice with PBS and stained using Alexa Fluor 488 phalloidin (Invitrogen, Cat. No. A12379) and DAPI (Thermo Scientific™, Cat. No. D1306). Fluorescence imaging was performed using confocal laser scanning microscopy to observe morphological changes.

### 5.14 Spheroid proliferation in GC

Spheroid viability was assessed using the AlamarBlue™ Cell Viability Reagent (Invitrogen, Cat. No. DAL1100). During the 5-day culture period on agarose molds, media supernatants were collected on days 1, 3, and 5. For the assay, 10 μL of each supernatant sample was mixed with 90 μL of AlamarBlue reagent in a 96-well plate. Absorbance was measured at 570 nm using a microplate reader.

### 5.15 Quantification of VEGF

During the 5-day culture of spheroids on agarose molds in a 24-well plate, culture media supernatants were collected on days 1, 3, and 5 for quantification of VEGF using ELISA. The VEGF ELISA kit (ABclonal, Cat. No. RK00023) was used according to the manufacturer's instructions. The 96-well plates provided in the kit were pre-coated with recombinant human VEGF (capture reagent). Each well was washed three times with assay washing diluent (400 μL well^−1^), and 100 μL of either VEGF standards or experimental samples was added. The standard curve ranged from 0 to 1000 pg mL^−1^. After incubation at room temperature for 2 h, pates were washed three times with wash buffer (400 μL well^−1^). Next, 200 μL of peroxidase-conjugated anti-VEGF polyclonal antibody (detection reagent) was then added to each well and incubated for 1 h at room temperature. After a subsequent wash step (3 washes, 400 μL well^−1^ each), streptavidin-HRP (100 μL well^−1^) was added and incubated for 30 min at room temperature. Following three more washes (400 μL well^−1^ each), the peroxidase-specific substrate was added (100 μL well^−1^). After approximately 20 min, stop solution was added (50 μL well^−1^) to terminate the peroxidase reaction, yielding a color intensity proportional to the VEGF concentration. The VEGF concentration was quantified by measuring absorbance at 450 and 540 nm using a microplate reader.

### 5.16 Quantification of spheroid diameter

Throughout the 5-day culture period of spheroids on agarose molds in a 12-well plate, images of the spheroids were captured daily using a microscope. The measurement region for diameter analysis was indicated by the yellow dotted circle in the bright-field image shown in Fig. S9a. Using this region of interest, the Feret diameter of each spheroid was meticulously measured with ImageJ software, allowing precise quantification and analysis of spheroid size over time and enabling a detailed assessment of spheroid growth and morphological changes.

### 5.17 HUVEC tube formation assay using conditioned media

Human umbilical vein endothelial cells (HUVECs; Lonza, Cat. No. 2519A) were used to assess the pro-angiogenic effects of spheroid-GC systems. HUVECs were cultured in EGM-2 medium (Lonza, Cat. No. CC-3162) and seeded onto 24-well plates at 5 × 10^4^ cells per well. Conditioned media were collected after 24 h from the following groups: VEGF-free control medium (-VEGF), VEGF-supplemented medium (+VEGF; 50 ng/mL recombinant human VEGF; PeproTech, Cat. No. 100-20-1 MG), spheroids only, spheroid +5% (w/v) GC, and spheroid + 7% (w/v) GC. HUVECs were incubated with 1 mL of each conditioned medium for 24 h at 37 °C, and tube-like structures were imaged using an inverted microscope. Total tube length, number of meshes, number of junctions, and total mesh area were quantified using ImageJ (Angiogenesis Analyzer plugin).

### 5.18 Anti-apoptotic and cytoprotective effects under oxidative stress

Salivary gland epithelial cells were seeded in 6-well plates at 5 × 10^5^ cells per well and cultured overnight. Oxidative stress was induced by treating cells with 500 μM H_2_O_2_ in complete medium for 24 h. For paracrine co-culture, spheroid and spheroid + GC (5% or 7% w/v GCs) were placed in Transwell inserts (0.4 μm pore size) above the cell monolayer, and cells were further cultured for 24 h in the continued presence of H_2_O_2_. Cells were then harvested, washed with cold PBS, and stained with an Annexin V-FITC/propidium iodide apoptosis detection kit according to the manufacturer's instructions. Flow-cytometric analysis was performed using a BD FACSCanto™ Clinical Flow Cytometry System (BD Biosciences, USA), and the proportions of live, early apoptotic, and late apoptotic/necrotic cells were quantified.

### 5.19 Macrophage culture, LPS stimulation, and ELISA analysis of M2/M1 markers

RAW 264.7 murine macrophages were seeded at a density of 3 × 10^4^ cells per well in 48-well plates and allowed to adhere overnight. Cells were then stimulated with lipopolysaccharide (LPS, final concentration 1 μg/mL) (Sigma-Aldrich, Cat. No. L6529) for 24 h to induce M1 polarization, after which the LPS-containing medium was replaced with the respective conditioned media as described above. Following 24 h treatment with conditioned media, culture supernatants from RAW 264.7 cells were collected and centrifuged to remove debris, and the levels of the M1-associated cytokine TNF-α were measured using a mouse TNF-α ELISA kit (Mouse TNF-alpha ELISA Kit, RK00027) according to the manufacturer's instructions. The M2-associated cytokine IL-10 was quantified using a mouse IL-10 ELISA kit (BMS614, Mouse IL-10 Coated ELISA, 96 tests). Absorbance was read at the recommended wavelength using a microplate reader, and cytokine concentrations were calculated from standard curves. The relative M2/M1 polarization ratio was determined by dividing IL-10 levels by TNF-α levels and normalizing to the LPS-only group.

### 5.20 IR-damaged SG mouse model

*In vivo* studies were conducted with the approval of the Institutional Animal Care and Use Committee (No. 24-0049-S1A0) at Seoul National University Hospital, Seoul, Republic of Korea. C57BL/6 mice (male, 6 weeks old, weighing 16–20 g; Orientbio, Seongnam, Republic of Korea) were utilized for the experiments. The mice were anesthetized *via* intraperitoneal injection of Zoletil 50 (tiletamine + zolazepam, 0.006 mL/10 g; Virbac, Hamilton, New Zealand) and Rompun (xylazine, 0.004 mL/10 g; Bayer, Leverkusen, Germany). To induce IR-induced SG hypofunction, the head and neck regions were exposed to a single 15 Gy dose of 6-MV X-rays using a 21EX-S system (Varian, UK) at a dose rate of 250 MU/min. One day post-IR, various treatments were prepared, including 1% (w/v) gelatin solution, 7% (w/v) gelatin, spheroids suspended in 1% gelatin solution, 7% (w/v) GC, spheroids encapsulating 5% (w/v) GC, and spheroids encapsulating 7% (w/v) GC, and injected into the submandibular gland (SMG) tissues. For the GC-incorporated groups, the injected gels were crosslinked with blue light (450 nm) using a Dental LED Rainbow Curing Light Lamp (Resin Cure Plastic Handle, 3 Mode; Cat. No. 1070800717) for 1 min immediately after injection. To ensure consistent exposure to blue light, the remaining groups without GC were also irradiated under the same conditions. The mice were randomly divided into seven experimental groups: Group 1: Sham (1% w/v gelatin solution; n = 5); Group 2: 15-Gy IR + 1% (w/v) gelatin injection (n = 5); Group 3: 15-Gy IR + 7% (w/v) gelatin injection (n = 5); Group 4, 15-Gy IR + AdMSC spheroids in 1% (w/v) gelatin (n = 7); Group 5: 15-Gy IR + 7% (w/v) GC injection (n = 7); Group 6: 15-Gy IR + spheroids encapsulating 5% (w/v) GC (n = 7); and Group 7: 15-Gy IR + spheroids encapsulating 7% (w/v) GC (n = 7). The AdMSC spheroid group was injected with 70 spheroids, each with a diameter of 300 μm, using a Scalp Vein Set (Doowon Meditec). Body weight was monitored following IR, and SMG tissues were harvested for analysis at 1 week and 6 weeks post-injection.

### 5.21 In vivo tracking of DiR-labeled AdMSC spheroids by IVIS imaging

AdMSCs were labeled with the near-infrared fluorescent dye DiR (DiIC18(7); DiR′; Invitrogen, Cat. No. D12731) prior to spheroid formation. Cells at 70–80% confluence were washed with PBS and incubated in serum-free medium containing 1 μM DiR for 20 min at 37 °C, followed by three washes with PBS to remove unbound dye. The DiR-labeled AdMSCs were then detached, resuspended in growth medium, and seeded into agarose-based micro-molds for spheroid formation as described above. After 3 days of culture, DiR-labeled spheroids (approximately 300 μm in diameter) were collected from the molds, pooled, and either injected directly or encapsulated within GCs and injected into the IR-damaged mouse SG according to the *in vivo* protocol.

For *in vivo* fluorescence imaging, mice were anesthetized and scanned using an IVIS imaging system (IVIS Lumina S5, PerkinElmer) at predetermined time points (1, 3, 5, 7, 10, 14, 21, and 28 days) with excitation/emission settings appropriate for DiR (typically Ex 748 nm/Em 780 nm). Fluorescent signals in the SG region were quantified as average radiant efficiency using the instrument software with a fixed region of interest applied to all groups and time points. At day 28, whole-body imaging was followed by ex vivo imaging of major organs (heart, liver, spleen, lung, kidney and stomach).

### 5.22 Immunohistochemical analyse

Formalin-fixed, paraffin-embedded tissue sections (4-μm thick) were prepared for histological analysis. Sections were subjected to the following staining protocols to evaluate tissue morphology and composition: H&E (VECTOR Hematoxylin QS Nuclear Counterstain, Cat. No. H3404; Sigma-Aldrich, Cat. No. E40095G) was used to visualize general tissue structure and cellular morphology. Alcian Blue and Periodic Acid-Schiff (ScyTek Laboratories, Cat. No. APS2) was performed to detect mucopolysaccharides and glycoproteins, highlighting the presence of acidic mucins. MT staining (ScyTek Laboratories, Cat. No. TRM1) was used to differentiate between collagen and muscle tissue, facilitating the evaluation of fibrosis and connective tissue architecture. All staining protocols were performed using conventional methods to ensure accurate and reproducible results.

### 5.23 Collagen content assay

For quantitative assessment of collagen content, 10 mg of SG tissue was used per sample. Tissues were homogenized and processed using a colorimetric collagen assay kit (DG-COL100; DoGenBio, Korea) according to the manufacturer's instructions. After acid hydrolysis and subsequent reaction with the supplied detection reagents, absorbance was measured at the specified wavelength using a microplate reader, and collagen concentration was calculated from a standard curve and normalized to tissue weight (μg collagen per mg tissue).

### 5.24 Immunofluorescence analyses

Paraffin-embedded tissue sections (4 μm) were deparaffinized, rehydrated, and subjected to antigen retrieval by boiling in citrate buffer (Sigma-Aldrich, Cat. No. C9999) for 1 h. Slides were cooled to room temperature for 20 min and then placed on ice for another 20 min. Tissue sections were permeabilized using 0.5% Triton X-100 (Sigma-Aldrich, Cat. No. X100) and blocked with 2.5% Normal Horse Serum Blocking Solution (VECTOR, Cat. No. S012) for 30 min. Sections were incubated with primary antibodies against AQP5 (1:200, Abcam, ab78486) for acinar cells, α-SMA (1:200, Abcam, ab7817) for myoepithelial cells, MIST1 (1:50, Abcam, ab187978) for acinar cells, SOD2 (1:100, Abcam, ab13534), a ROS scavenging marker, 8-OHdG (1:50, Santa Cruz, sc393871), an oxidized DNA damage marker, NOX4 (1:100, GeneTex, GTX121929), an oxidative stress marker, CD31 (1:40, Novus Biologicals, NB600), an endothelial cell marker for ECs, VEGF-A (1:100, Abcam, ab52917) for angiogenesis marker, CD86 (1:100, Abcam, ab220188) for M1 marker, CD163 (1:200, Abcam, ab182422) for M2 marker, and cleaved caspase-3 (1:100, Cell Signaling Technology, S9661), an apoptosis marker. Following primary antibody incubation, appropriate Alexa Fluor 594-conjugated and Cy5-conjugated secondary antibodies were used for detection. Nuclei were counterstained with DAPI (Thermo Scientific, Cat. No. D1306). Immunofluorescent images were captured using the EVOS M700 microscope (Invitrogen).

### 5.25 Quantitative real-time PCR for angiogenesis-related genes

Total RNA was isolated from SG tissue using TRIzol™ LS Reagent (Invitrogen, Cat. No. 10296010) according to the manufacturer's protocol. Complementary DNA (cDNA) was synthesized from 500 to 1000 ng of total RNA using PrimeScript RT Master Mix (TaKaRa, Cat. No. RR036A), and quantitative PCR was performed with TB Green® Premix Ex Taq™ (TaKaRa, Cat. No. RR420A) on a real-time PCR system using gene-specific primers for VEGF, HGF, CD31, and vWF, with GAPDH as the internal reference gene. Relative mRNA expression levels were calculated using the 2^ΔΔCt^ method and normalized to the sham control group.

### 5.26 Statistical analysis

All statistical analyses were performed using Origin Pro 2021B software (Data Analysis Software, Northampton, MA, USA) and GraphPad Prism 8 (GraphPad Software, La Jolla, CA, USA). Specifically, statistical evaluations for Fig. 2, Fig. 3, Fig. 4, Fig. 5 and Figs. S1, S2, S8, and S11 were conducted using Origin Pro 2021B, while Fig. 6, Fig. 7, Fig. 8, Fig. 9, Fig. 10, Fig. 11, Fig. 12 and Figs. S9, S14, and S15 were analyzed using GraphPad Prism 8. Data are presented as mean ± standard deviation. Comparisons between two groups were conducted using unpaired t-tests. For comparisons among multiple groups, one-way analysis of variance (ANOVA) followed by Tukey's post-hoc test was applied. Prior to applying these parametric tests, normality of the data distribution was assessed using the Shapiro-Wilk test. If the data were found to be non-normally distributed, appropriate non-parametric test (Kruskal-Wallis test) were used instead. Statistically significant differences are indicated as follows: ns (not significant), ∗*P* ≤ 0.05, ∗∗*P* ≤ 0.01, and ∗∗∗*P* ≤ 0.001.

## CRediT authorship contribution statement

**Byulhana Kim:** Writing – review & editing, Writing – original draft, Visualization, Validation, Methodology, Investigation, Formal analysis, Data curation. **Hyerin Yoo:** Writing – review & editing, Writing – original draft, Visualization, Validation, Investigation, Formal analysis, Data curation. **Young Ju Son:** Writing – review & editing, Validation, Methodology, Investigation, Data curation, Conceptualization. **Seyun Ahn:** Investigation, Formal analysis. **Jeonghoon Lee:** Investigation. **Young Kim:** Methodology, Investigation. **Tae Hee Kim:** Writing – review & editing, Methodology, Investigation. **Min Rye Eom:** Investigation, Data curation. **Young Bin Choy:** Supervision, Project administration, Funding acquisition. **Justin J. Chung:** Writing – review & editing, Writing – original draft, Visualization, Validation, Supervision, Project administration, Methodology, Investigation, Funding acquisition, Conceptualization. **Seong Keun Kwon:** Writing – review & editing, Writing – original draft, Validation, Supervision, Resources, Project administration, Methodology, Investigation, Funding acquisition, Conceptualization.

## Data availability statement

The data that support the findings of this study are available from the corresponding author upon reasonable request.

## Ethics approval statement

In vivo studies were conducted with the approval of the Institutional Animal Care and Use Committee (No. 24-0049-S1A0) at Seoul National University Hospital, Seoul, Republic of Korea.

## Declaration of interest statement

Justin J. Chung is an Early Career Editorial Board Member for Bioactive Materials and was not involved in the editorial review or the decision to publish this article. All authors declare that there are no competing interests.
