# Supplementary material for: AdMSC spheroids encapsulating antioxidant hybrid protein carrier for irradiation-damaged salivary gland repair
Source: Bioact Mater. 2026 Apr 3;63:166–87. doi: 10.1016/j.bioactmat.2026.03.049 (PMC13088972; doi:10.1016/j.bioactmat.2026.03.049)
Supplement: Multimedia component 1 [file mmc1.docx]

Supporting Information

AdMSC spheroids encapsulating antioxidant hybrid protein carrier for irradiation-damaged salivary gland repair

**
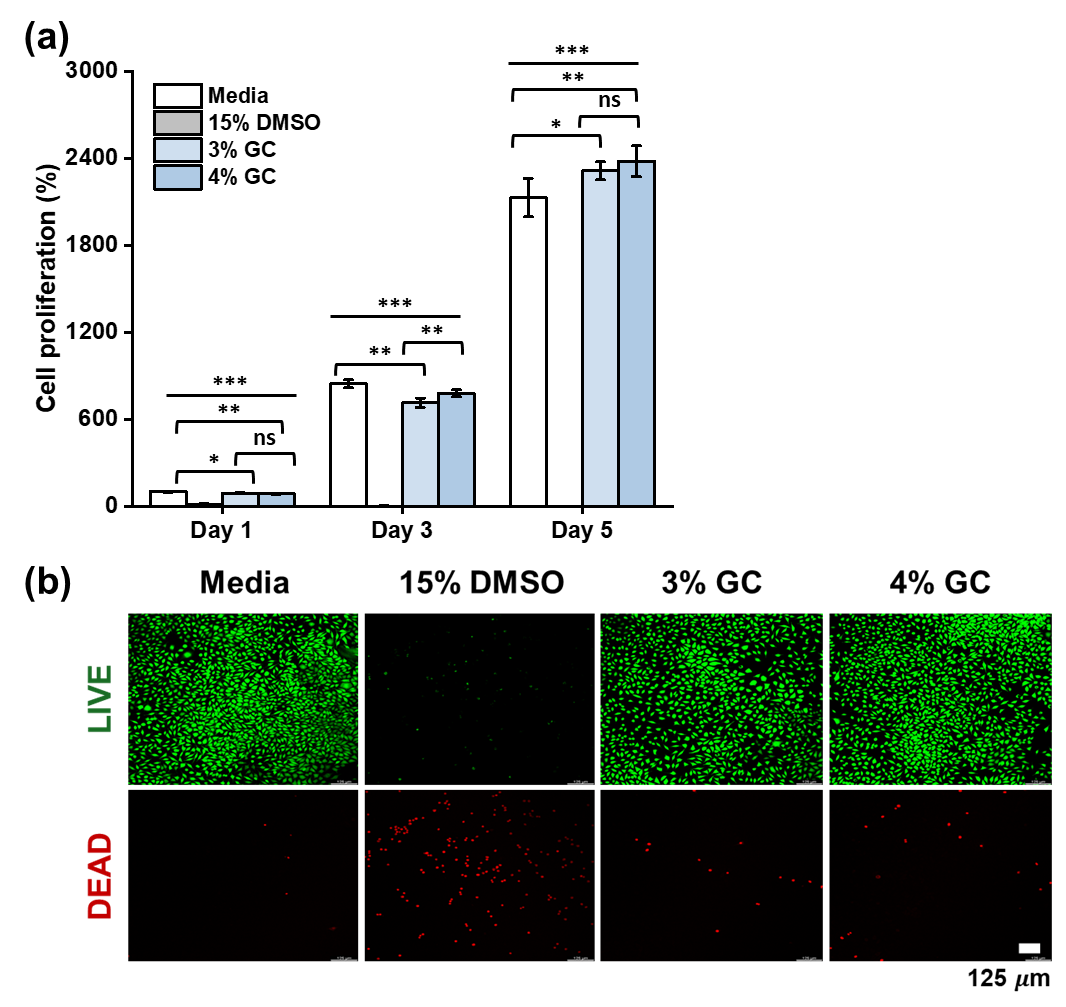
**

Fig. S1. Biocompatibility of 3% and 4% (w/v) GCs as cell carriers. (a) L929 cell proliferation following incubation with extracts of 3–4% (w/v) GCs for 1, 3, and 5 d (n = 5). (b) Confocal images of L929 cells stained with Live (green) and Dead (red) reagents after 3 d of incubation in the 3–4% (w/v) GC extracts. Data indicate mean $\boldsymbol{\pm}$ SD (n = 5). Statistical analysis was performed using one-way ANOVA followed by Tukey’s post-hoc test. ^ns^P > 0.05, ^*^P $\boldsymbol{\leq}$ 0.05, ^**^P $\boldsymbol{\leq}$ 0.01, and ^***^P $\boldsymbol{\leq}$ 0.001. Scale bar = 125 $\boldsymbol{\mu}$m.


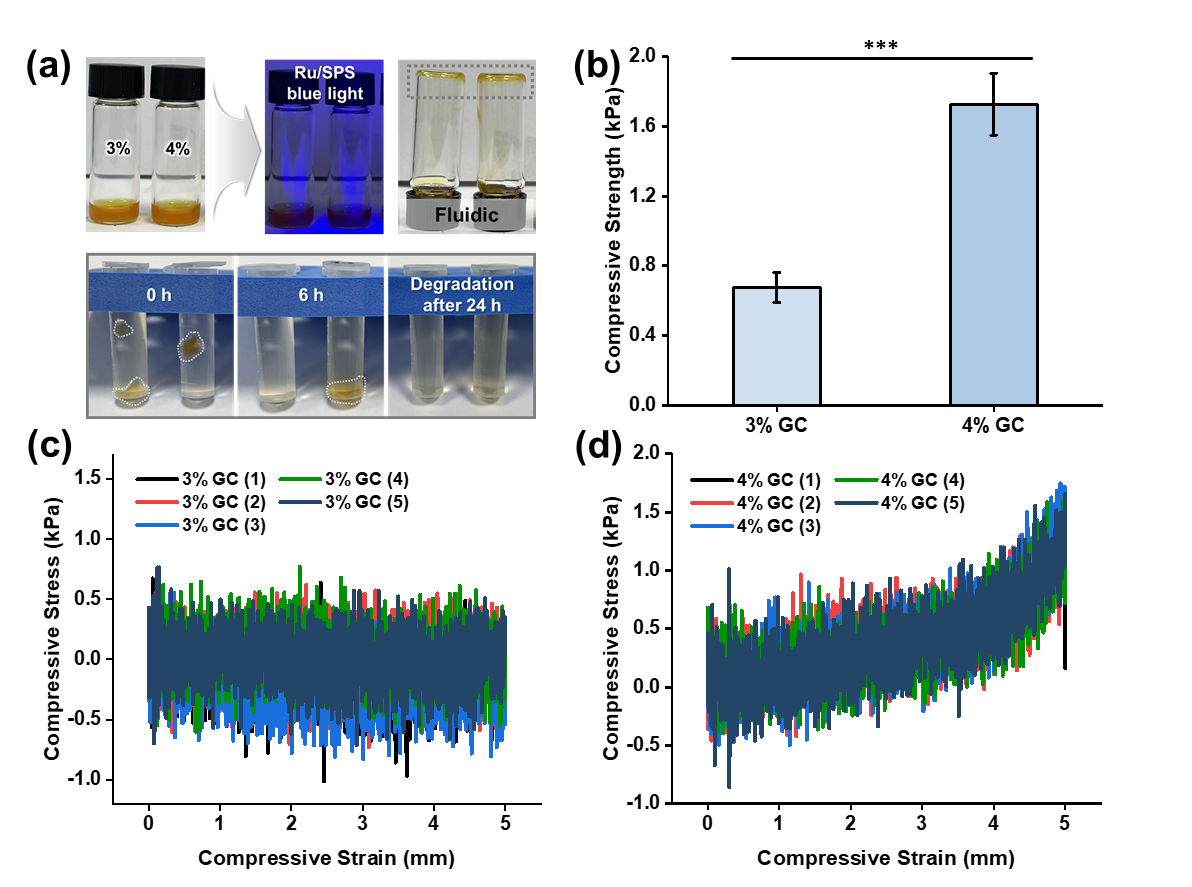


Fig. S2. Inadequate mechanical properties of 3% and 4% (w/v) GCs for spheroid encapsulation. (a) Representative images showing the low structural stability of 3% and 4% GCs after photo-crosslinking using Ru/SPS and blue light, followed by degradation after 24 h in 1X PBS at 37 °C. (b) Ultimate compressive strength (kPa) of 3% and 4% (w/v) GCs. (c, d) Representative compressive stress-strain curves for both concentrations. Data indicate mean $\boldsymbol{\pm}$ SD (n = 5). Statistical analysis was performed using one-way ANOVA followed by Tukey’s post-hoc test. ^ns^P > 0.05, ^*^P $\boldsymbol{\leq}$ 0.05, ^**^P $\boldsymbol{\leq}$ 0.01, and ^***^P $\boldsymbol{\leq}$ 0.001.


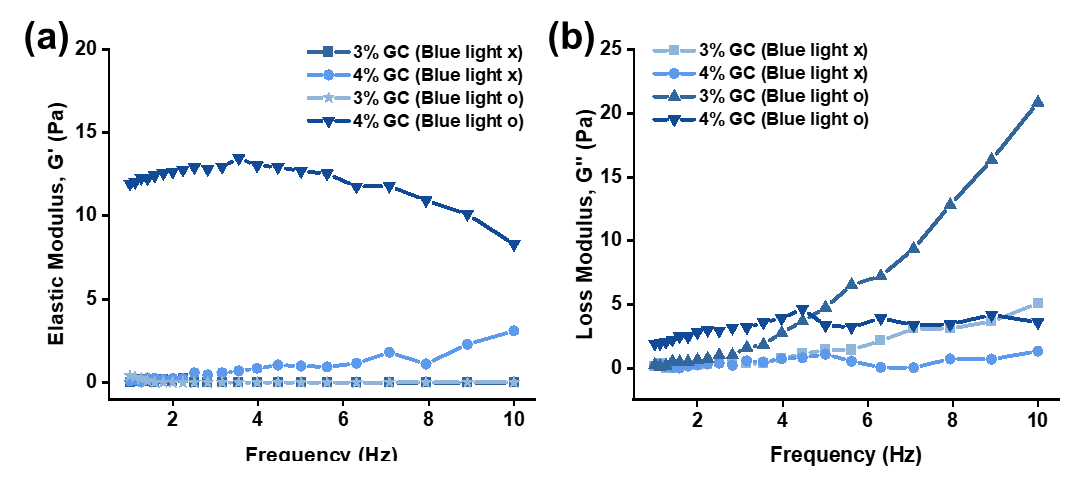


Fig. S3. Inadequate rheological properties of 3% and 4% (w/v) GCs for spheroid encapsulation. (a) Storage modulus (G’) and (b) loss modulus (G’) of 3% and 4% (w/v) GCs, with and without photo-crosslinking using Ru/SPS and blue light system.


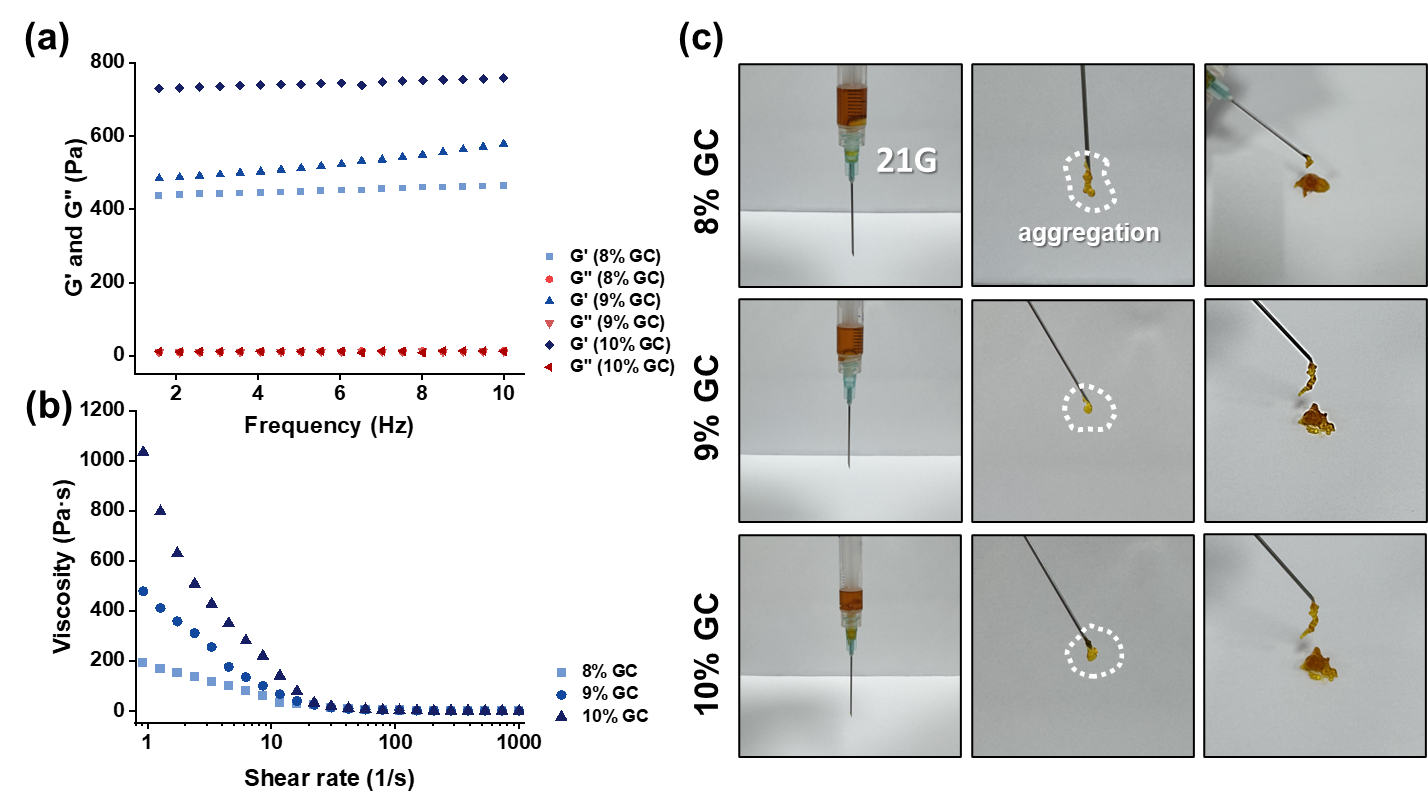


Fig. S4. Inadequate rheological properties and poor injectability of 8-10% (w/v) GCs. (a) Storage modulus (G’) and loss modulus (G’) of 8-10% (w/v) GCs. (b) Shear-thinning behavior of 8-10% (w/v) GCs. (c) Representative optical images demonstrating the poor injectability and 21G needle clogging of 8-10% (w/v) GCs.


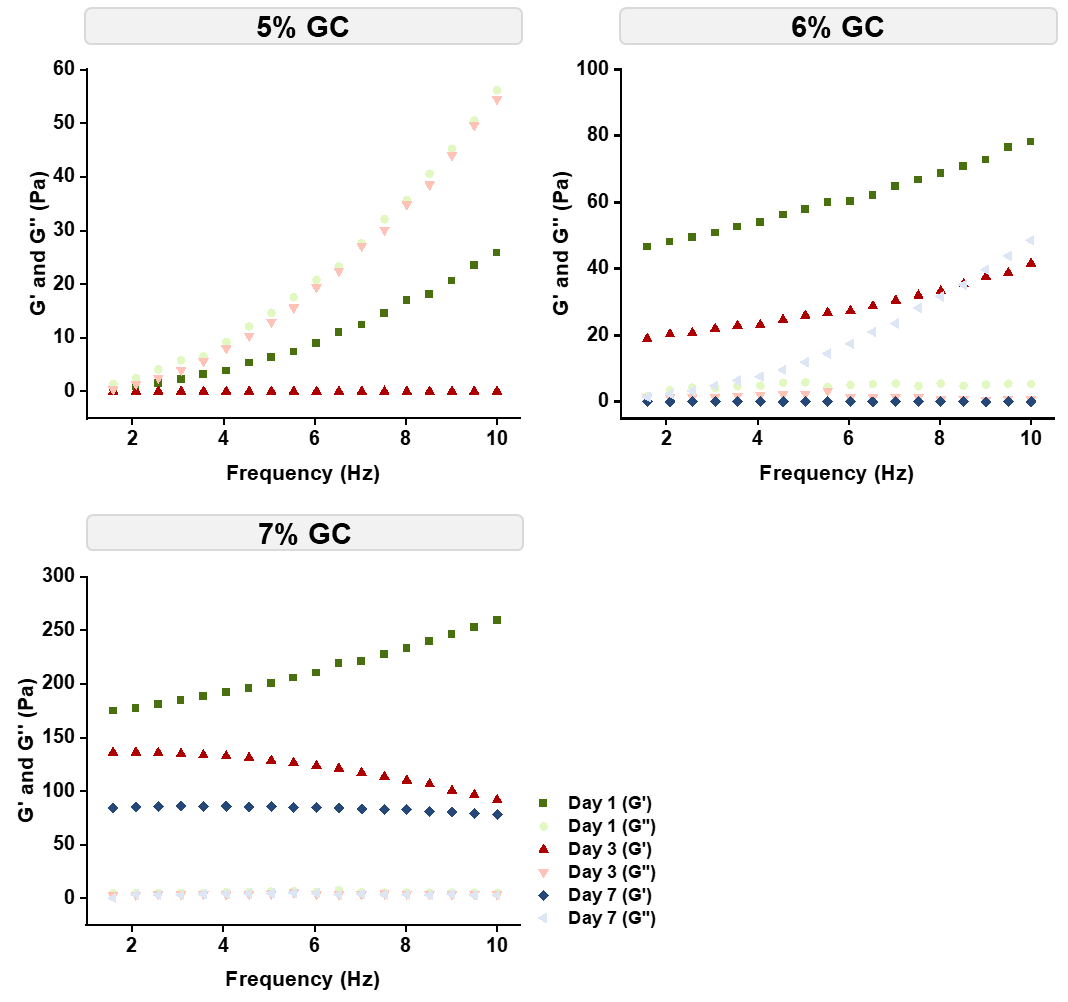


Fig. S5. Time-dependent rheological stability of 5–7% (w/v) GCs under physiological conditions. The frequency sweep analysis (1-10 Hz) of 5-7% (w/v) GCs were evaluated during incubation in 1X PBS (pH 7.4) at 37 ^o^C for 7 days.


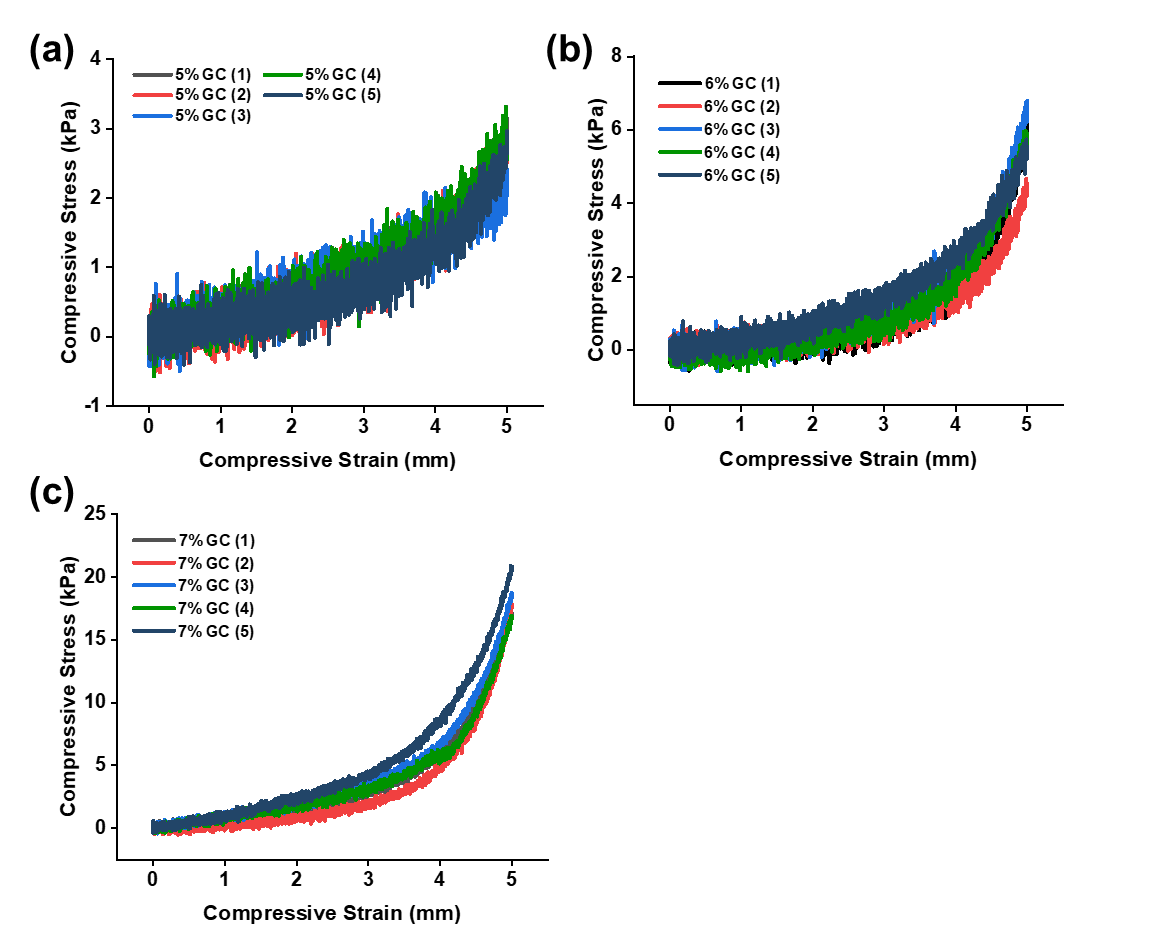


Fig. S6. Compressive stress-strain curves of 5–7% (w/v) GCs. (a) Compressive stress-strain curve of 5% (w/v) GC, (b) 6% (w/v) GC, and (c) 7% (w/v) GC.


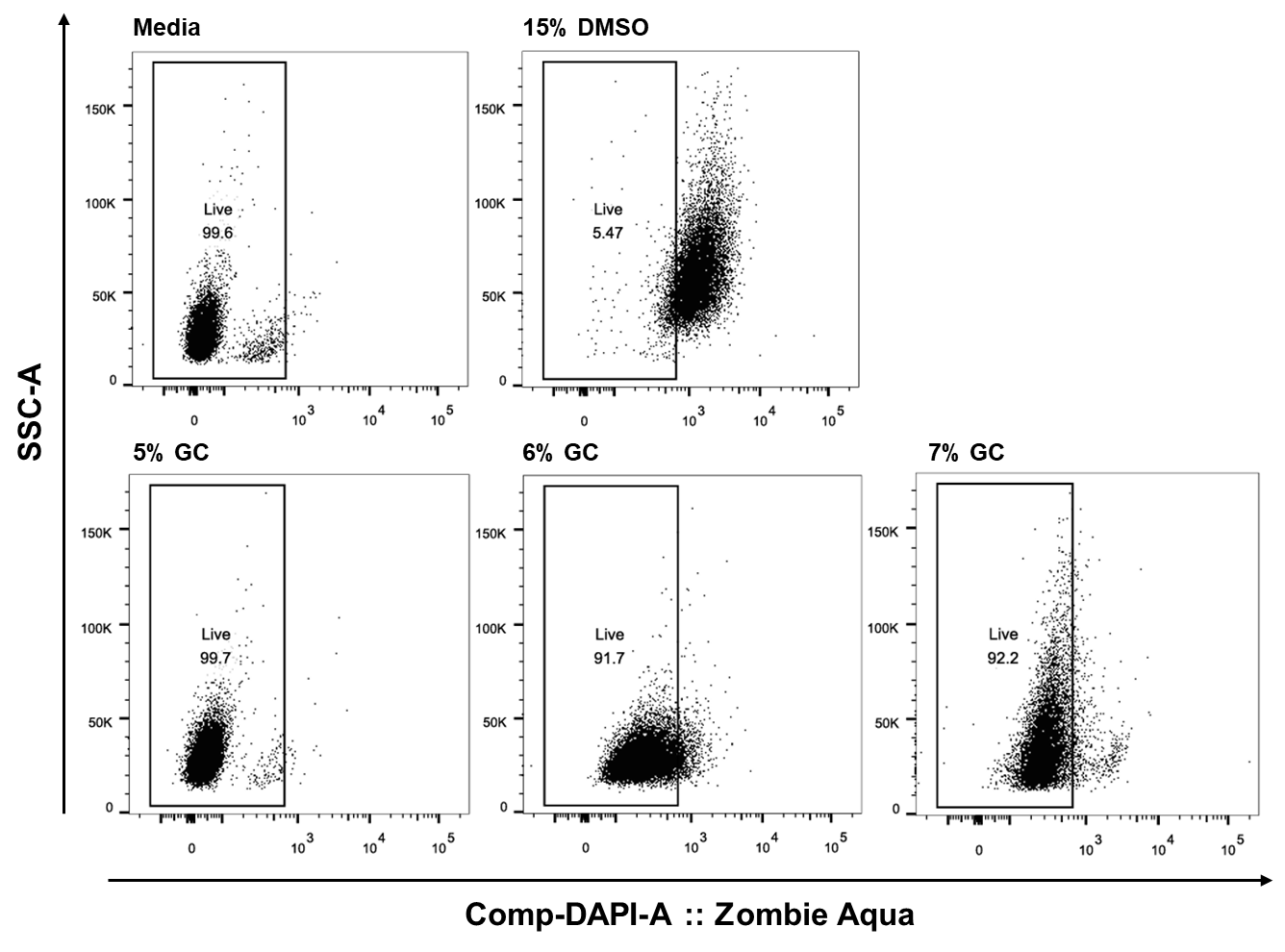


**Fig S7. Cytotoxicity assessment of GC using flow cytometry.** Representative flow cytometry dot plots showing the viability of L929 cells treated with 5-7% (w/v) GC for 3 days. Cells were stained with Zombie Aqua (DAPI-A channel) to distinguish live (Zombie Aqua-negative) from dead (Zombie Aqua-positive) populations.


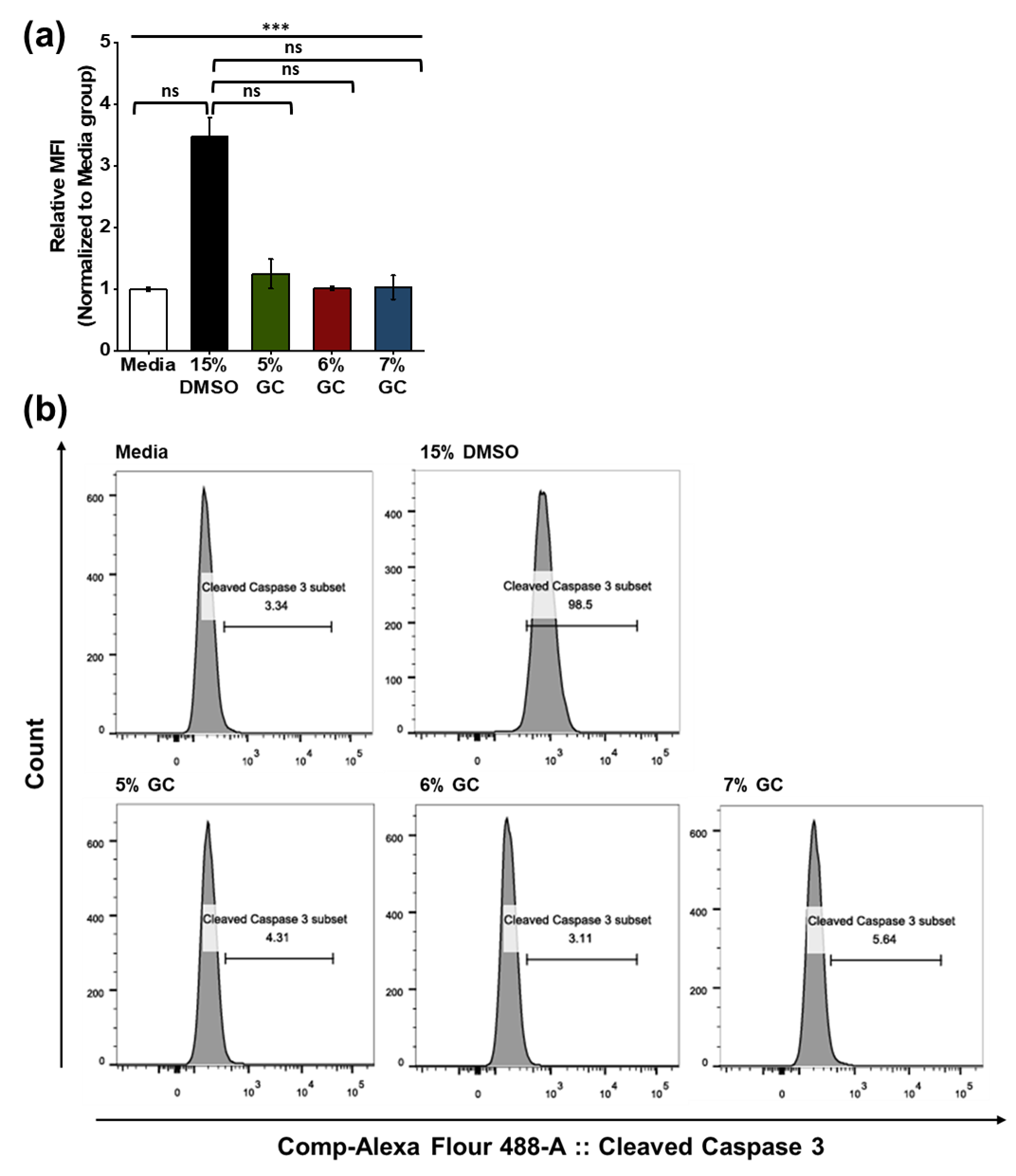


**Fig S8. Analysis of apoptosis via cleaved caspase 3 expression.** (a) Quantitative analysis of relative mean fluorescence intensity (MFI), normalized to the Media group. (b) Histogram plots reveal that the percentage of cleaved caspase3. Data presented as mean $\pm$ SD (n = 5). Statistical analysis was performed using one-way ANOVA followed by Tukey’s post-hoc test. ^ns^ P > 0.05, ^*^ P$\leq$ 0.05, ^**^P $\leq$ 0.01, and ^***^P $\leq$ 0.001.


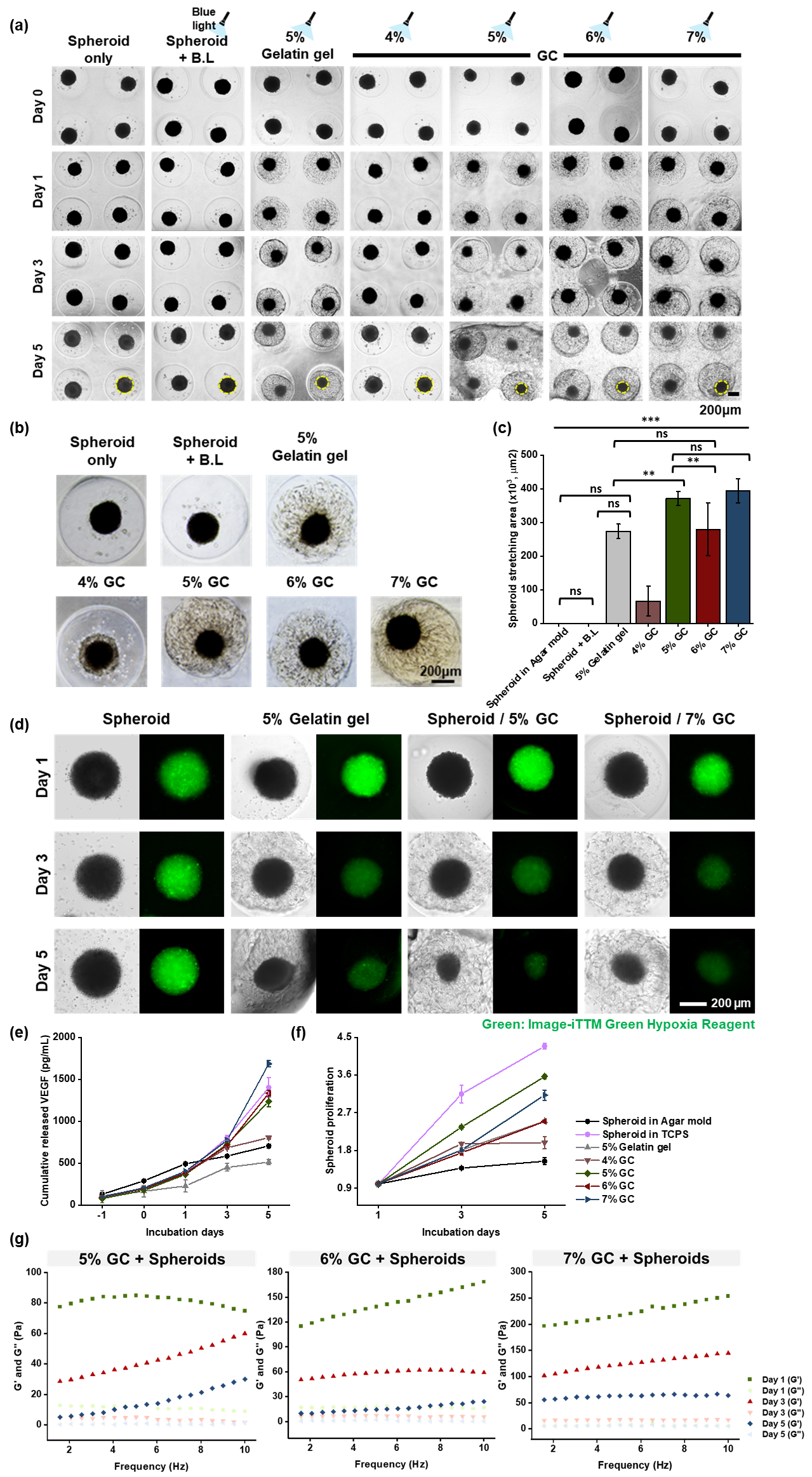


**Fig. S9. Morphology and functional changes of AdMSC spheroids loaded in hybrid protein cell carriers.** (a) Representative phase-contrast images showing morphological changes and radial spreading of AdMSC spheroids cultured for up to 5 days under different conditions: spheroid only, spheroid exposed to blue light (spheroid + B.L), 5% (w/v) gelatin gel, and 4-7% (w/v) GCs. Yellow dotted lines indicate the spheroid boundary used for area measurement. Scale bar: 200 μm. (b) Magnified images of spheroids at day 3 under each condition to compare the extent of cell outgrowth from the spheroid core. (c) Quantification of spheroid spreading area at day 3 for each condition. (d) Time-dependent hypoxic area in spheroids cultured with or without hydrogel carriers. Representative phase-contrast and fluorescence images show spheroids encapsulating 5% (w/v) gelatin gel, 5% (w/v) GC, or 7% (w/v) GC and stained for hypoxia using Image-iT Green Hypoxia Reagent on days 1, 3, and 5. Green fluorescence indicatets hypoxic regions within the spheroids, allowing visualization of changes in the extent of the hypoxic areas over time under each culture condition. Scale bar = 200 $\mu$m. (e) Cumulative VEGF secretion profiles over time from spheroids cultured in agarose molds (3D), on tissue culture polystyrene (TCPS; 3D‑to‑2D transition), in 5% (w/v) gelatin, and in 4-7% (w/v) GCs. At day 5, TCPS and GC groups (4–7% w/v) showed significantly higher cumulative VEGF release than agarose spheroids, and 7% (w/v) GC displayed the highest VEGF level with significant differences compared with most other conditions (***p < 0.001, **p < 0.01, *p < 0.05). (f) Spheroid proliferation over 1, 3, and 5 days under the same conditions, quantified by Alamar Blue assay. At day 5, TCPS and GC groups (4–7% w/v) exhibited significantly greater proliferation than agarose spheroids, with 5% (w/v) GC showing the highest proliferation and significant differences relative to 5% (w/v) gelatin and 4% (w/v) GC (***p < 0.001, **p < 0.01, *p < 0.05). Data are presented as mean ± SD (n = 3). (g) Frequency-dependent storage (G′) and loss (G′′) moduli of spheroid encapsulating 5-7% (w/v) GCs at days 1, 3, and 5 of incubation. Data are presented as mean $\pm$ SD. Statistical analysis was performed using one-way ANOVA followed by Tukey’s post-hoc test.


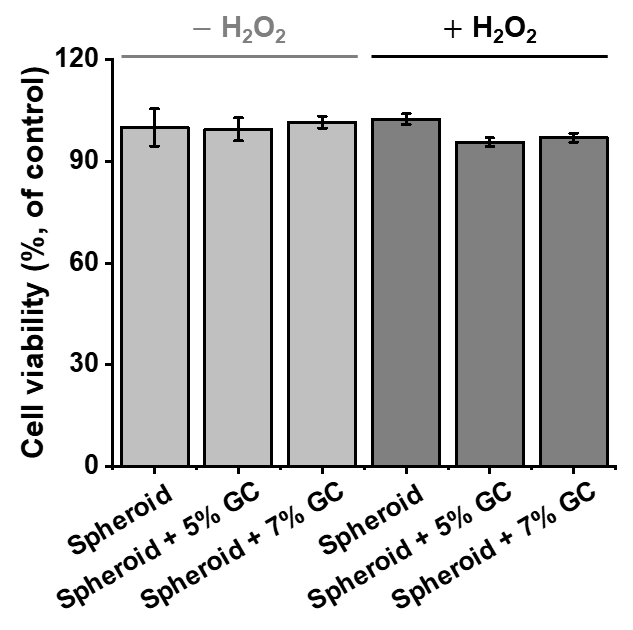


**Fig. S10. Viability of AdMSC spheroids under ROS conditions with or without GCs.** Spheroids were cultured alone (Spheroid) or encapsulated in 5% or 7% (w/v) GCs, and exposed to either basal conditions ($-$H_2_O_2_) or oxidative stress induced by H_2_O_2_ ($+$H_2_O_2_). Cell viability was evalulated using the Alamar Blue assay and expressed as a percentage relative to the corresponding untreated control group (mean $\pm$ SD), and even in the absence of GC (Spheroid only), spheroids exhibited resistance to oxidative stress without significant loss of viability, indicating preservation of their functional capacity in the ROS-rich environment.


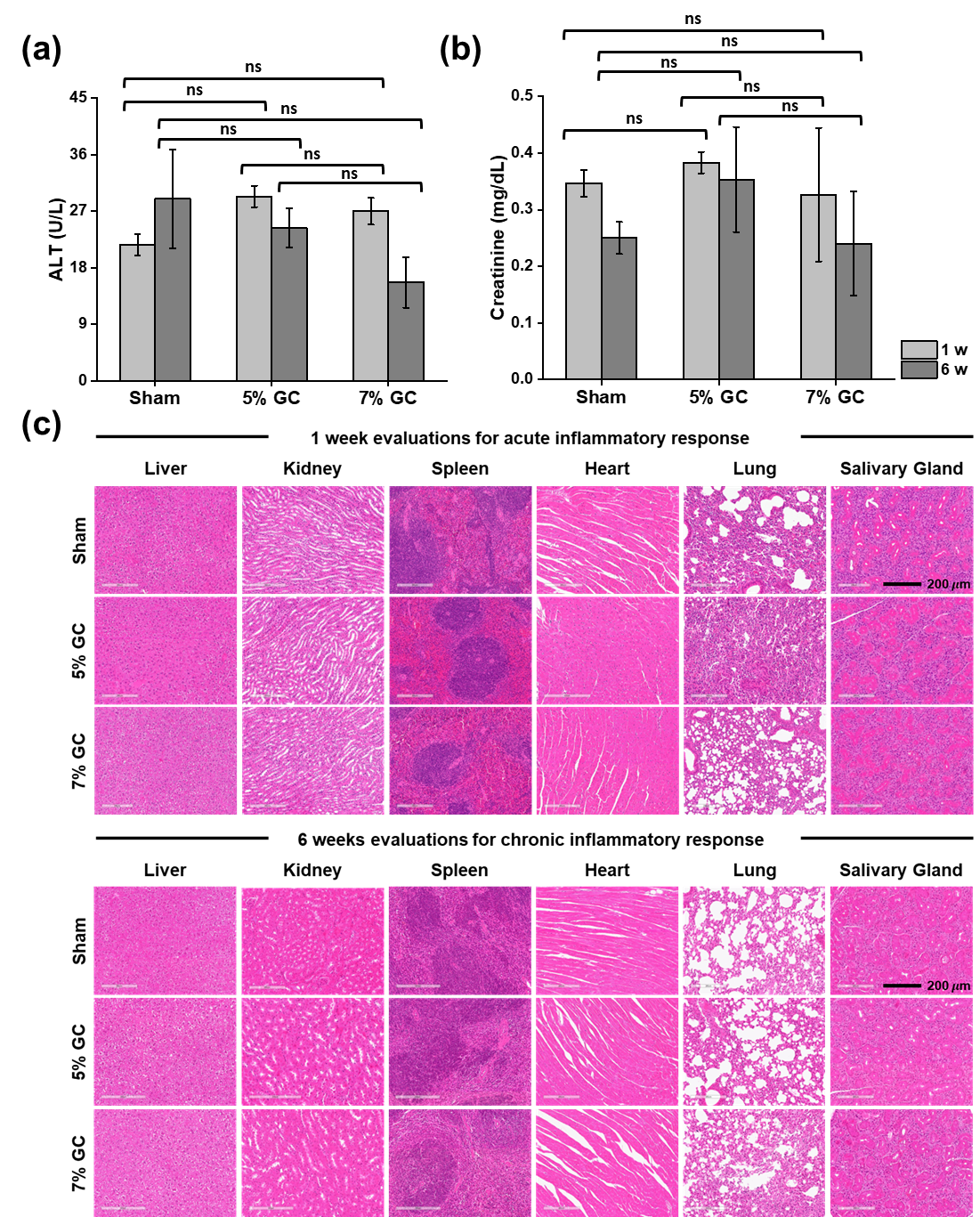


Fig. S11. In vivo biocompatibility and systemic assessment of GC. Biochemical analysis of serum markers for organ function. (a) Alanine aminotransferase (ALT) for liver function and (b) Creatinine for kidney function. (c) Representative H&E-stained images of major organs (liver, kidney, spleen, heart, lung, and salivary gland) at 1 week (acute inflammatory response) and 6 weeks (chronic inflammatory response) post-treatment. Data presented as mean $\boldsymbol{\pm}$ SD (n = 3). Statistical analysis was performed using one-way ANOVA followed by Tukey’s post-hoc test. ^ns^ P > 0.05, ^*^ P$\boldsymbol{\leq}$ 0.05, ^**^P $\boldsymbol{\leq}$ 0.01, and ^***^P $\boldsymbol{\leq}$ 0.001.


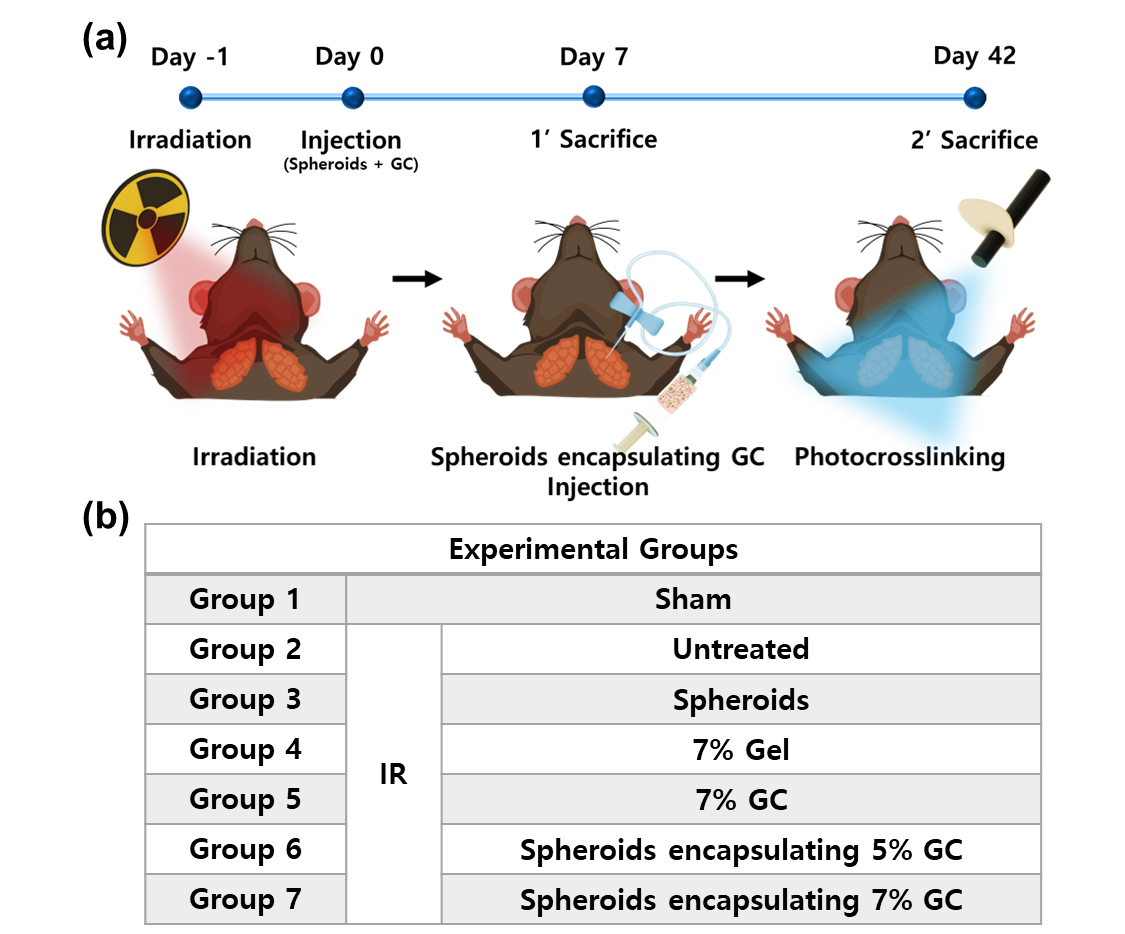


Fig. S12. In vivo study design. (a) Schematic timeline of the in vivo experimental procedure: Day -1: IR; Day 0: injection of control or treatment formulations; Days 7 and 42: sample collection and mouse sacrifice. (b) Group allocation table for in vivo studies.


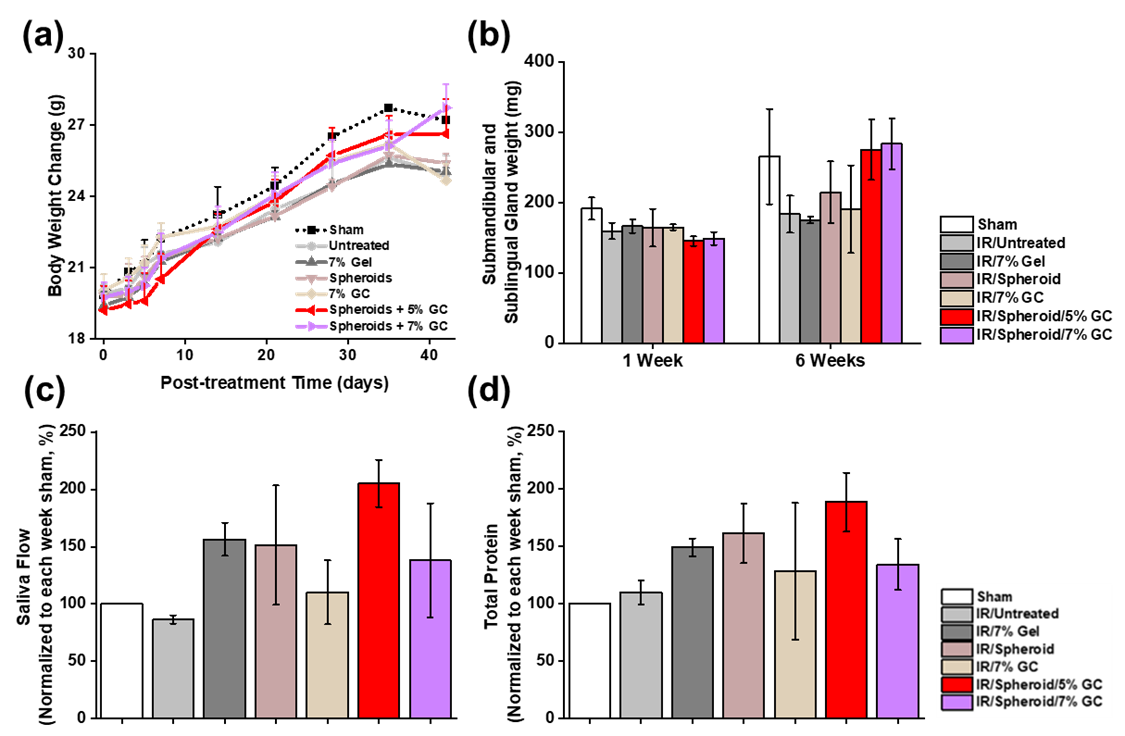


Fig. S13. Functional outcomes of spheroids encapsulating GC for IR-damaged salivary glands. (a) Body weight changes over 42 d post-treatment. Group 7 (spheroids in 7% GC) showed a significant weight increase from day 7, surpassing even the sham group by day 21, indicating systemic recovery. (b) Salivary gland (SG) weights at 1 and 6 weeks post-IR. Group 6 and group 7 showed significantly higher gland weights at 6 weeks than irradiated controls. (c) Normalized saliva flow rate at 6 weeks demonstrated substantial functional recovery in groups 6 and 7. (d) Total protein content in collected saliva, reflecting restored secretory function, was also elevated in treatment groups. These results indicate that the combination therapy enables sustained functional regeneration of irradiated salivary glands. Although statistical comparisons among treatment groups (4–7) did not reach significance (ns), likely due to the limited sample size and intrinsic inter-animal variability in the in vivo model, the consistent trends across body weight, gland weight, saliva flow, and total protein support superior functional recovery in the spheroid + GC groups.

**
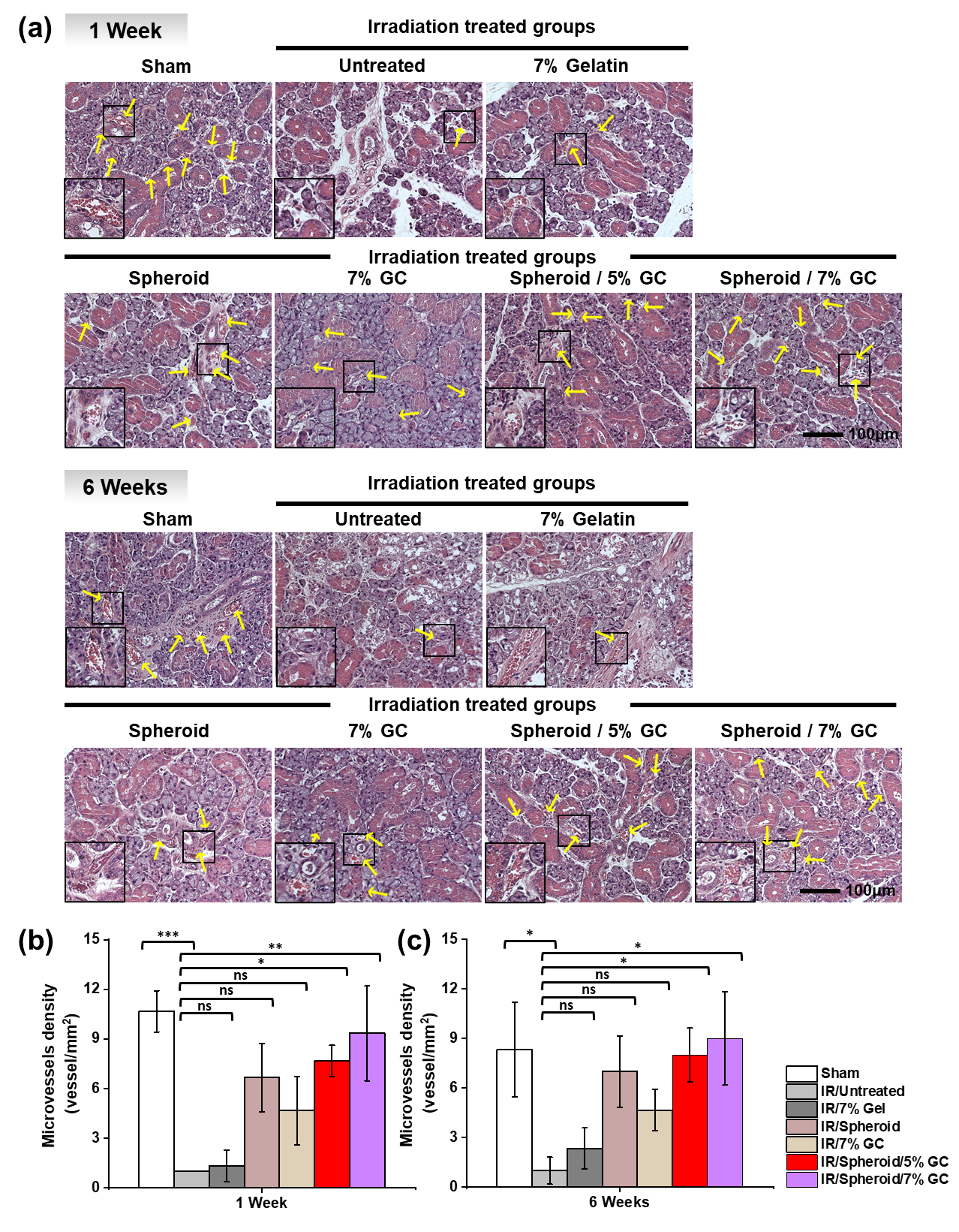
**

Fig. S14. Density of microvessels. (a) Representative H&E‑stained SG tissue sections, with Yellow arrows indicate microvessels, and inset black boxes indicate regions that are shown at higher‑magnification as small black boxed areas within each field. (scale bar: 100 μm). (b, c) Bar graphs show the quantified microvessel density (vessels/mm²) at 1 week and 6 weeks. Data presented as mean ± SD (n = 3). Statistical analysis was performed using one-way ANOVA followed by Tukey’s post-hoc test. ns P > 0.05, * P ≤ 0.05, **P ≤ 0.01, and ***P ≤ 0.001.


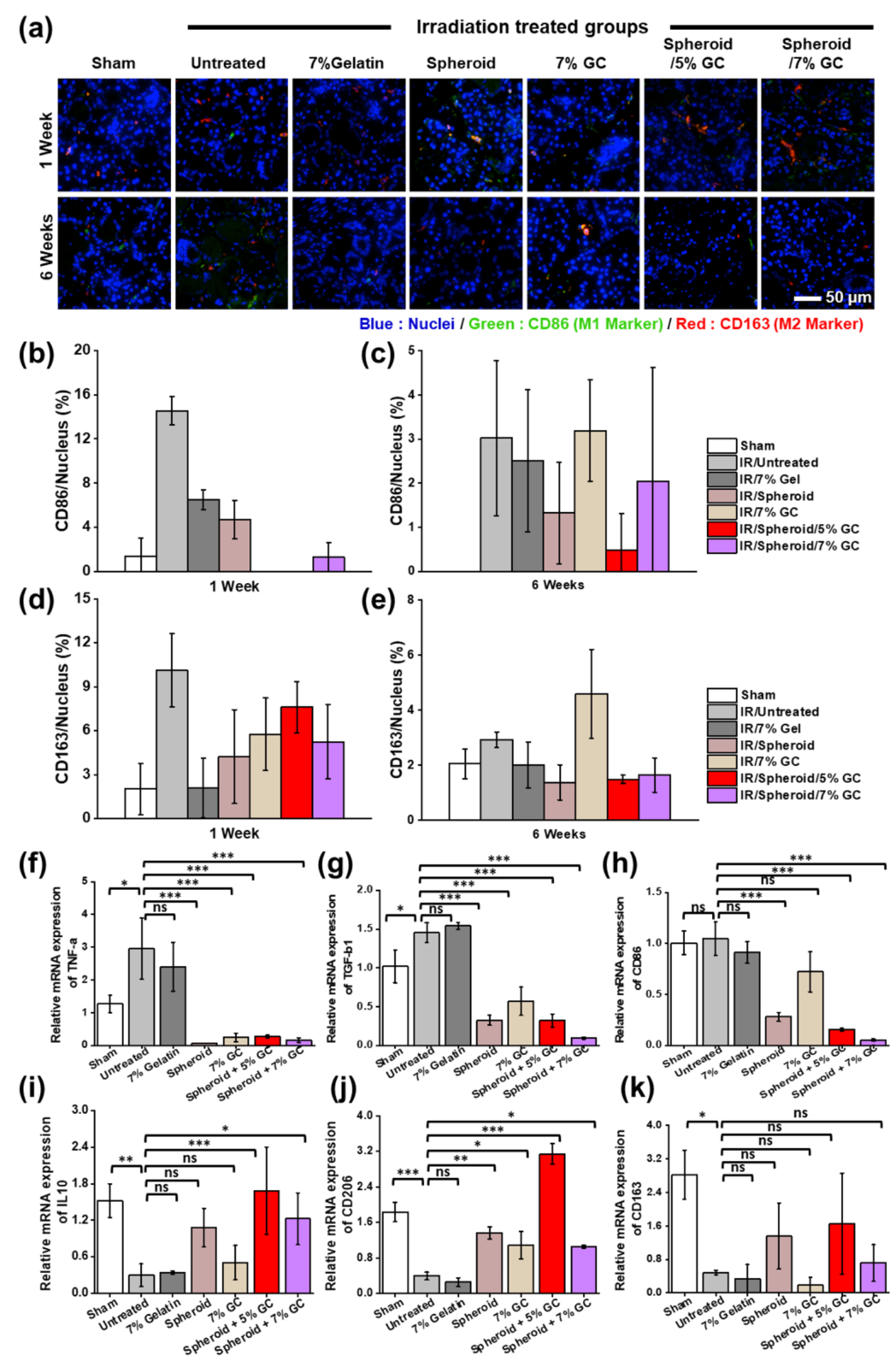


Fig. S15. Immune response modulation by spheroids encapsulating GC in IR-damaged mouse salivary glands (SGs). (a) Representative immunofluorescence images of SG tissue sections at 1 and 6 weeks post-IR. Nuclei were stained with DAPI (blue), M1 macrophage marker CD86 (green), and M2 macrophage marker CD163 (red). (b–e) Quantitative analysis of CD86⁺ and CD163⁺ macrophage populations at each time point. Percentages of CD86⁺/nuclei (%) and CD163⁺/nuclei (%) were determined from immunofluorescence images. At 1 week post-IR, group 2 exhibited pronounced CD86⁺ cell infiltration, indicating a strong pro-inflammatory (M1) response. In contrast, treatment with spheroids encapsulated in GC significantly reduced the M1 population and increased M2 polarization, with the effect being most prominent in group 7. These immunomodulatory trends were maintained through week 6, suggesting a long-term shift toward a tissue-regenerative immune environment. Quantitative real-time PCR analysis of M1-associated markers (f) TNF-α, (g) TGF-β1, and (h) CD86, and M2-associated markers (i) IL10, (j) CD206, and (k) CD163 in salivary gland tissues harvested at 1 week after irradiation. Salivary glands were collected at 1 week, lysed, and processed for RNA extraction and qPCR; 6-week samples were not analyzed by qPCR because host remodeling at later time points was expected to generate heterogeneous cell populations and confound interpretation of early immunomodulatory effects.^[1-2]^ Relative mRNA expression levels were normalized to a housekeeping gene and are presented as fold change relative to the Sham group. Data presented as mean $\boldsymbol{\pm}$ SD (n = 5). Statistical analysis was performed using one-way ANOVA followed by Tukey’s post-hoc test. ^ns^ P > 0.05, ^*^ P$\boldsymbol{\leq}$ 0.05, ^**^P $\boldsymbol{\leq}$ 0.01, and ^***^P $\boldsymbol{\leq}$ 0.001.

Reference

[1] M. L. Novak, T. J. Koh, *J Leukocyte Biol* **2013**, 93, 875.

[2] J. Rojas, J. Salazar, M. S. Martínez, J. Palmar, J. Bautista, M. Chávez-Castillo, A. Gómez, V. Bermúdez, *Scientifica* **2015**, 2015.
